# The Inter-Relationship between Dietary and Environmental Properties and Tooth Wear: Comparisons of Mesowear, Molar Wear Rate, and Hypsodonty Index of Extant Sika Deer Populations

**DOI:** 10.1371/journal.pone.0090745

**Published:** 2014-03-06

**Authors:** Mugino Ozaki Kubo, Eisuke Yamada

**Affiliations:** 1 The University Museum, The University of Tokyo, Tokyo, Japan; 2 Department of Earth and Environmental Science, Graduate School of Science and Engineering, Kagoshima University, Kagoshima, Japan; University of Arkansas, United States of America

## Abstract

In reference to the evolutionary trend of increasing cheek tooth height in herbivorous ungulates, the causes of dental abrasion have long been debated. Interspecific comparisons of extant ungulates have revealed that both phytoliths in grass and external abrasive matter may play important roles. Using analysis of extant sika deer living in various environments and showing continuous latitudinal variation in food habits from northern grazing to southern browsing, we quantitatively evaluated the influence of dietary and environmental properties on three dental variables: mesowear score (MS), molar wear rate, and M_3_ hypsodonty index. We used 547 skulls and 740 mandibles from 16 populations of sika deer to obtain the dental measurements. We found that only graminoid proportion in diet correlated with MS and the molar wear rate, implying that phytoliths in grass abrade dental tissues. In contrast, annual precipitation in habitat was not correlated with any of the dental variables. We also found a significant correlation between the molar wear rate (selective pressure for high-crowned molars) and the M_3_ hypsodonty index of extant sika deer, implying an evolutionary increment in molar height corresponding to the molar wear rate. Our intraspecific comparative analyses provide further support for use of mesowear analysis as a paleodiet estimation method; it not only reveals staple food types (graminoids or dicots) but also implies regional or seasonal variation in the diet of the species.

## Introduction

Living herbivorous ungulates (hooved animals) show distinct feeding styles, which can be classified into three categories: grazers that predominantly consume graminoids, browsers that consume leaves of dicotyledons and/or fruits, and intermediate (or mixed) feeders that vary their diet between grazers and browsers [1]. Previous studies have shown clear associations between feeding style and morphological features, especially in cheek teeth [2]–[4]. Because graminoids contain more phytoliths (silica) than browses [5] and because plants growing in open and dry environments are adhered by soil and grit more often than those growing in closed and wet environments [6], grazers and/or species in open habitats may ingest more abrasive matter than browsers and/or species in closed habitats [7]. There has been a long debate on which abrasive matter (i.e., intrinsic silica or extrinsic grit) plays a more prominent role in tooth wear [4], [7]–[12], though the role of phytoliths has been challenged by recent experimental studies [9], [12]. Regardless of the origin of the abrasive matter, the amount of abrasives that animals ingest will have a close relationship with cheek teeth morphologies and characteristics, and this relationship has been used to reconstruct the paleoecology of extinct animals. Three characteristics of cheek teeth have been widely investigated: (1) relative height of molars (hypsodonty index), (2) microscopic wear on the tooth enamel surface (microwear), and (3) macroscopic tooth wear (mesowear). The hypsodonty index is a measurement obtained by dividing tooth crown height by width or length [13], [14]. This index has been reported to be associated with diet and habitat type; species consuming a larger amount of graminoids and living in drier habitats have larger hypsodonty indices to counteract life-long dental abrasion [3], [7], [13], [15], [16]. Microwear is a microscopic scar left on the tooth enamel surface and can be observed under a scanning electron microscope or a stereomicroscope [17]–[20]. Patterns of microwear differ with feeding habits; grazers have more striations than browsers, corresponding to the larger amount of abrasiveness in their diets [18], [21], [22]. Mesowear analysis examines the relative facet development of cheek teeth [23]. In this analysis, tooth occlusal relief is categorized as “high” or “low” and the cusp shape as “sharp,” “rounded,” or “blunt” through observation with the naked eye or a hand lens. Though the categorization of occlusal wear is subjective, previous studies showed that interobserver error did not significantly affect results [24], [25]. Using comparative data on living ungulates, it was shown that browse-dominant diets promote attrition (tooth–tooth contact) and result in sharp cusps with high occlusal relief, whereas grass-dominant diets promote abrasion (tooth–food contact) and result in blunt cusps with low occlusal relief [23]. Mesowear analysis allows a non-destructive examination of a large number of specimens in a short time and at a low cost. Because of these advantages, this method has been applied to paleoecological reconstruction with increasing frequency [26]–[32].

Hypsodonty, microwear, and mesowear reflect dental wear at different time scales [23]. Evolution of hypsodont cheek teeth is considered a species-specific adaptation to increased dental wear; thus, the hypsodonty index can track ecological changes that occurred over geological time [33]. However, microwear on the tooth surface reflects the properties of food eaten by an animal a few days before its death, which is problematic in species that change their diets seasonally (“Last Supper Effect”) [34], [35]. Mesowear variables have been shown to be stable when juvenile and senescent animals are excluded, and this implies that the mode of tooth wear tends to be stable, except for the earliest and latest life stages, and thus is ideal for reconstructing average diets of animals during their lifetime [23], [36]. The process of dental wear is understood to relate to the evolution of hypsodonty as follows: (1) abrasive matter, either intrinsic or extrinsic, remove occlusal tooth enamel and dentine microscopically (appearing as striations on the enamel), (2) accumulation of microscopic scars results in blunting of cusps (appearing as low occlusal relief and rounded or blunt cusps in mesowear analysis), and (3) more rapid wear of dental tissues (enamel, dentine, and cement) results in shorter functional duration of teeth, finally bringing about the evolution of hypsodonty over geological time (appearing as a high hypsodonty index). In some circumstances other selective pressures may also bring about evolution of hypsodonty; for example, adaptive change in life history traits toward elongation of longevity, which is considered to be applicable to insular ruminants without predators, may require longer durability of dentition [37]–[39]. If the abovementioned causality between abrasive diet and evolution of hypsodonty is hold among herbivorous ungulates in general, a close inter-relationship among dental variables can be expected. Until recently, however, the relationship has not been investigated. Kaiser et al. [40] were the first to investigate the relationships between the hypsodonty index, mesowear, and ecological factors (percentage of grass in natural diets, habitat type, and annual precipitation in habitats) using 75 extant ungulate species. As expected, it was shown that mesowear and the hypsodonty index were significantly correlated. Correlation with diet or habitat environment differed for the two variables; the hypsodonty index was significantly correlated with both diet and habitat environment, whereas mesowear was significantly correlated with diet but not habitat environment. On the basis of these results, Kaiser et al. [40] hypothesized that dietary properties are responsible for characterizing cusp shape, while external abrasives originating from the habitat environment do not influence cusp shape but accelerate the tooth wear rate. The different responses of mesowear and the hypsodonty index to dietary and environmental properties indicate that additional data on the tooth wear rate, a variable linking mesowear and the hypsodonty index, are needed to clarify the inter-relationship.

Another important topic that has not been extensively investigated is mesowear within species. This topic contains two separate questions: (1) whether populations within a species, which differ in diet or habitat environments, differ in mesowear and (2) whether species with greater intraspecific dietary variation show greater variation in mesowear than those with lesser dietary variation. A pioneering study on the former question was conducted by Kaiser and Schulz [41], supplemented by Schulz and Kaiser [42], which tested the relationship between habitat environment and mesowear among extant wild populations, although they did not quantitatively test the effect of diet. Recently, one of the authors (EY) performed a preliminary analysis using four sika deer populations with distinct food habits and found significant differences in mesowear variables between the populations [43]. If diet is influential in shaping molar cusps, as suggested by Kaiser et al. [40], then quantitative investigation should reveal a relationship between diet and mesowear among sika deer populations. The latter question on the relationship between dietary variation and mesowear variation among species has yet to be tested. The ability to infer dietary variation in extinct species from mesowear would significantly benefit research into paleoecological reconstruction.

Sika deer (*Cervus nippon*) in Japan are ideal species to quantitatively test correlations among the dental variables related to tooth wear, i.e., mesowear, tooth wear rate, and hypsodonty index, as well as correlations between diet, habitat environments, and dental variables. Sika deer show a north–south variation in diet, from northern grazing populations to southern browsing populations [44]. Since there are abundant rainfall in sika deer habitats (annual precipitation is above 1000 mm), these deer inhabits in forests, offering a valuable opportunity to assess the effect of grass on dental wear without confounding habitat factors. Sika deer populations differ in the molar wear rate, which tends to correlate with graminoid consumption and annual precipitation in their habitat, although neither correlation was statistically significant [45]. We here presented additional data on mesowear, the molar wear rate, and the hypsodonty index of sika deer to test correlations with ecological factors, as well as among the dental variables.

The aims of the present study were as follows. (1) To exploit the utility of mesowear in estimating the dietary properties of extinct species, we investigate intraspecific relationship between mesowear and ecological variables in sika deer, and investigated the relationship between dietary variation and mesowear variation both within and among species. (2) To clarify the most influential factor determining dental wear and whether and how it relates to the evolution of hypsodonty, we tested the relationship between dental variables and ecological factors as well as among the three dental variables on the basis of comparisons of sika deer populations.

## Materials and Methods

### Sika deer populations

**Figure 1:**
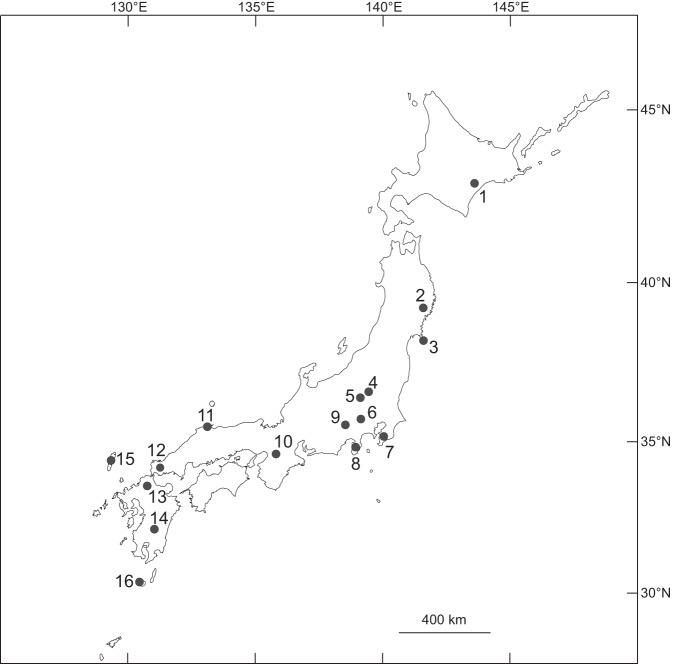
Map of the sika deer (*Cervus nippon*) populations studied in the Japanese archipelago. The numbers correspond to those in Column 1 in Table 1.

Sixteen sika deer populations were studied (Table 1, Fig. 1). We used museum collections for investigation. The institutes which housed sika deer skulls were also presented in Table 1 and the specimen information was provided in Table S1. Sika deer in the northern areas inhabit mixed conifer–broad-leaved forests or deciduous broad-leaved forests and consume a large amount of dwarf bamboo (e.g., *Sasa nipponica*), a graminoid that dominates the forest understory. In contrast, deer in the south–west inhabit evergreen broad-leaved forests and rely more heavily on browses [44]. Procedures for collecting dietary data are described in detail below. Annual precipitation levels for each area were collected from the Japan Meteorological Agency (http://www.data.jma.go.jp). Most adult skull specimens were aged by histological investigation of the cementum annuli of lower first incisors [46], [47].

**Table 1:** Ecological characteristics of the sika deer (*Cervus nippon*) populations.

Population	Major vegetation	Annual precipitation (mm)	Graminoids %		Non-graminoids %	Fruits %	Literature on diets	
			Mean	SD*				Institute which house sika deer skulls^**^
1. Eastern Hokkaido	Deciduous broad-leaved and mixed forest	1119	46.3	19.6	50.4	2.9	Yokoyama et al. [64]	UMUT, TPM
2. Mt. Goyo	Coniferous or deciduous broad-leaved forest	1507	52.5	12.7	28.5	0	Takatsuki and Ikeda [65]	UMUT
3. Kinkazan Island	Deciduous broad-leaved forest with some open grasslands	1099	65.8	12.6	29.5	4.6	Padmalal and Takatsuki [66]	UMUT
4. Nikko	Deciduous broad-leaved forest	2168	73.9	3.6	26.0	0.0	Takatsuki [67]	UMUT, TPM
5. Ashio	Deciduous broad-leaved forest with some open grasslands	1793	90.0	9.1	7.2	0.0	Koganezawa [54]	UMUT, TPM
6. Okutama	Deciduous broad-leaved forest	1652	32.1	2.6	63.0	4.8	Jiang unpublished data^****^	UMUT
7. Boso Peninsula	Evergreen broad-leaved forest	2027	45.2	8.3	50.4	4.3	Asada and Ochiai [68]	NHMIC
8. Izu Peninsula	Deciduous broad-leaved and evergreen broad-leaved forest	2715	30.5	NA^***^	69.4	0.1	Kitamura et al. [69]	UMUT
9. Yamanashi	Deciduous broad-leaved forest	2219	42.2	22.1	54.8	2.8	Jiang unpublished data	UMUT
10. Nara Park	Open grassland with planted conifers and deciduous trees	1391	81.4	11.6	18.6	0.0	Takatsuki and Asahi [70]	HUM
11. Shimane	Evergreen broad-leaved forest	1611	38.1	22.8	52.1	9.8	Shimane Prefectural Government [71]	UMUT
12. Yamaguchi	Evergreen broad-leaved forest	1723	6.5	3.0	86.2	6.0	Jayasekara and Takatsuki [72]	UMUT
13. Fukuoka	Evergreen broad-leaved forest	1585	7.2	5.6	79.5	0.8	Ikeda et al. [73]	UMUT
14. Mt. Shiraga	Evergreen broad-leaved forest	2794	25.6	NA^***^	78.5	0.3	Japan Wildlife Research Center [74]	FFPRI
15. Tsushima Island	Evergreen broad-leaved forest	1779	3.4	2.3	89.2	4.1	Suda [75]	TPM, HUM
16. Yakushima Island	Evergreen broad-leaved forest	4436	4.4	NA^***^	78.9	9.8	Takatsuki [76]	UMUT, TPM

^*^Standard deviation (SD) of graminoid proportion in diets was calculated from averages of four seasons. ^**^Abbreviations of the institutes are: UMUT, The University Museum, The University of Tokyo; TPM, Tochigi Prefectural Museum; NHMIC, Natural History Museum and Institute, Chiba; HUM, The Hokkaido University Museum; FFPRI; Forestry and Forest Products Research Institute. ^***^Seasonal data on diet were not provided. ^****^The ecological survey of Okutama deer was conducted by the Bureau of Waterworks, Tokyo Metropolitan Government.

### Mesowear data

**Figure 2:**
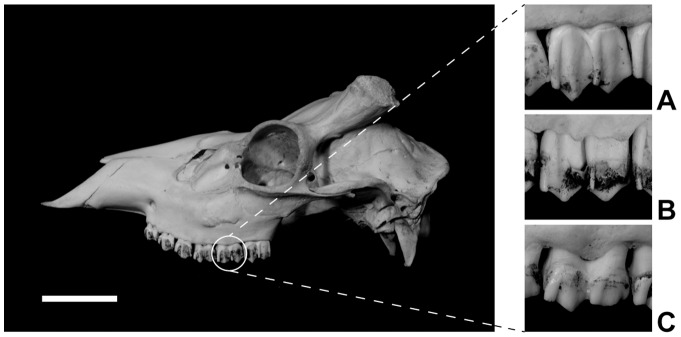
Examples of mesowear score (MS) of sika deer specimens (buccal view of left M_2_, teeth not to scale). (A) MS  = 0: high occlusal relief and sharp cusp shape (Tsushima Island, M398). (B) MS  = 1: high occlusal relief and round cusp shape (eastern Hokkaido, M212). (C) MS  = 2: low occlusal relief and round cusp shape (Nikko, M172). The scale bar below the skull is 5 cm.

We used 547 skull specimens of sika deer for mesowear analysis. The number of specimens examined for each population is presented in Table 2. Original mesowear data of individual specimens were provided in Table S1. Only adult specimens in which the third molar is in occlusion and in which the first molar retains an occlusal shape similar to the second molar were examined. Both female and male specimens were examined. Two mesowear variables (occlusal relief, OR: high or low; cusp shape, CS: sharp, round, or blunt) were recorded for the upper second molars (M^2^), as defined by Fortelius and Solounias [23]. All mesowear data were collected by a single author (EY) to avoid interobserver error. A mesowear score (MS) [30]–[32] was calculated for each sample according to the following scheme by Croft and Weinstein [27]: high relief and sharp cusps  = 0; high relief and round cusps  = 1; low relief and round cusps =  2; low relief and sharp cusps  = 2.5; high or low relief and blunt cusps  = 3 (Fig. 2). The mean and standard deviation of MS were calculated for each population.

**Table 2:** Mesowear score (MS), the molar wear rate, and the M_3_ hypsodonty index of sika deer.

Population	MS			MS (female only)		Molar wear rate		M_3_ hypsodonty index	
	N	Mean	SD	N	Mean	N	Mean	N	Mean
1. Eastern Hokkaido	21	0.62	0.50	10	0.60	98	−0.00816	11	1.78
2. Mt. Goyo	22	0.64	0.49	11	0.73	67	−0.00985	10	1.83
3. Kinkazan Island	124	0.88	0.66	86	0.79	48	−0.01161	3	1.88
4. Nikko	43	0.62	0.65	33	0.48	36	−0.01148	7	1.80
5. Ashio	7	1.00	0.82	6	0.83	24	−0.01310	–	–
6. Okutama	18	0.67	0.59	18	0.69	32	−0.00832	3	1.83
7. Boso Peninsula	81	0.47	0.50	44	0.50	66	−0.00904	11	1.89
8. Izu Peninsula	26	0.54	0.51	22	0.50	59	−0.00611	12	1.87
9. Yamanashi	15	0.67	0.62	9	0.56	20	−0.01116	5	1.82
10. Nara Park	31	0.48	0.68	12	0.58	38	−0.01112	2	1.81
11. Shimane	30	0.43	0.63	15	0.33	18	−0.00911	1	1.77
12. Yamaguchi	33	0.56	0.61	17	0.47	89	−0.00753	4	1.75
13. Fukuoka	20	0.45	0.51	13	0.46	28	−0.00615	3	1.68
14. Mt. Shiraga	–	–	–	–	–	82	−0.00723	12	1.81
15. Tsushima Island	56	0.07	0.26	32	0.03	35	−0.00458	3	1.67
16. Yakushima Island	20	0.30	0.47	9	0.33	–	–	2	1.82

Number of specimens used and mean and SD of MS are presented for each population.

For comparative purposes, we also calculated the mean and standard deviation of MS of 75 extant ungulates. The original data set from the study by Fortelius and Solounias [23] was supplemented by data from the study by Kaiser et al. [40] and three additional species (Japanese serow, *Capricornis crispus*; black wildebeest, *Connochaetes gnou*; and sitatunga, *Tragelaphus spekii*) collected for the present study.

### Molar wear rate and hypsodonty index of sika deer

We calculated the molar wear rate in each sika deer population on the basis of data from the study by Ozaki et al. [45], supplemented with additional six populations (Ashio, Okutama, Yamanashi, Nara Park, Shimane, and Tsushima Island). The number of specimens used to calculate the molar wear rate in each population is presented in Table 2. The total number of specimens which we measured molar height was 740. Same as that in the study by Ozaki et al. [45], only female specimens were used because of the scarcity of adult males, especially those over 5 years of age. Log-transformed lower first molar (M_1_) height, defined as the distance between the metaconid cusp tip and the cervical line, was regressed against age in months for each population, following the measurement and statistical methodologies described in the study by Ozaki et al. [45]. The resultant regression slope was defined as the molar wear rate. Because the number of measurable molars was largest for M_1_, followed by M_2_ and M_3_, the M_1_ wear rate is the most reliable estimate and can be used as a proxy of the tooth wear rate in general.

Data for the hypsodonty index of unworn molars were obtained from the study by Ozaki et al. [48], with additional specimens measured using the same measurement protocol. (1) Buccolingual cross-sectional images of the unworn (unerupted) molars were acquired using the microfocus X-ray CT scanning system at the University Museum, the University of Tokyo (TX225-ACTIS, TESCO Corporation, Tokyo, Japan), (2) heights and buccolingual breadths of molars were measured from the images, and (3) the height was divided by the buccolingual breadth to obtain the hypsodonty index. The detailed method is described in the study by Ozaki et al. [48]. We only measured female specimens. Because of the scarcity of unworn M_1_ and M_2_ specimens, we used only the hypsodonty index of M_3_ for the present analyses.

### Food habit data for sika deer and other extant ungulates

Food habit analyses have been performed previously for each sika deer population [44]. We collected dietary data from studies of stomach contents or fecal matter. We categorized the diets into the following three categories: graminoids (leaves, sheaths, and culms of graminoids), non-graminoids (leaves and flowers of non-graminoid plants such as trees, herbaceous plants, ferns and moss, twig, and bark of trees), and fruits (fruits, tubers, bulbs, storage organs, pods, and seeds). The percentage of each food item was obtained separately for each of the four seasons (spring, summer, autumn, and winter), if data collection was performed year-round; an annual mean was also calculated. For graminoid proportion, we also calculated the standard deviation of the annual mean, which represents the seasonal variability in consumption of graminoids, and defined it as “variability of graminoid consumption”.

Gagnon and Chew [49] reviewed the food habits of 78 extant African bovids on the basis of an extensive literature survey. They presented the proportion of three food types (“monocotyledons”, “dicotyledons”, and “fruits”) as a percentage and showed the seasonal and geographic variability in diet, as well as the reliability of the data. Following the data collection and summarization procedures described in the study by Gagnon and Chew [49], we supplemented their data with additional data from 37 species collected from 81 references (Table S2). Dietary information was categorized into three types: graminoids (equivalent to “monocotyledons” in the study by Gagnon and Chew [49]), non-graminoids (equivalent to “dicotyledons”), and fruits (equivalent to “fruits”). These definitions are concordant with those used for sika deer. If sources provided food type percentages on the basis of fecal or stomach content analysis or from feeding observations including bite counts or proportion of time spent, then data were scored as precise. When there were several sources, mean values were calculated and the data were scored as an average. In cases where percentages were incompletely reported, the data were estimated from explicit descriptions, and the data were scored as inadequate. The data were considered reliable if they were scored as precise or average. Moreover, dietary variation (caused by seasonal or geographic variation) was assigned as variable (yes or no). In contrast to the dietary data used in the study by Kaiser et al. [40], which was based on one reference per species in most cases and only presented the percentage of grass in natural diets, our synthetized data are based on a number of references with quantitative dietary data and present a more comprehensive picture of the dietary variation among living ungulate species.

### Statistical analyses

#### Relationship between dental variables and ecological variables

Using data from 16 sika deer populations, we first tested for differences of MS among populations by the Kruskal-Wallis test (non-parametric version of one-way analysis of variance), with MS of individual sika deer being the response variable and populations as the categorical variable. Next, we used population data for inquiring relationship between dental variables and dietary and environmental factors (i.e., percentage of each of the three food items and annual precipitation in habitat). First of all, we checked for normality of population mean of MS, because MS was an ordinal variable. We found that population mean of MS distributed normally (Shapiro-Wilk normality test, n = 15, *W* = 0.96, *P* = 0.67). Therefore, we adopted general linear model (GLM). The response variable in GLM was population mean of MS, molar wear rate or M_3_ hypsodonty index and the predictor variables were graminoid proportion, fruit proportion, annual precipitation and interaction terms between annual precipitation and the former two (hereafter referred as graminoid × annual precipitation and fruit × annual precipitation, respectively). We applied stepwise model selection based on Akaike information criterion corrected for small sample size (AICc) to select the best model predicting each dental variable [50].

To compare results between intraspecific and interspecific analyses, interspecific relationship between MS and dietary variables was investigated by GLM, with MS being the response variable and proportion of graminoids and fruits being the predictor variables. The same test was additionally conducted for restricted data set, in which we only included the species with selenodont dentition and adequate sample size (over 10 individuals) [51], [52]. Because there is an explicit linear relationship between graminoid and non-graminoid proportions both within and among species (among sika deer populations, N = 16, *r* = −0.97, *P*<0.0001; among ungulate species, N = 73, *r* = −0.84, *P*<0.0001), we excluded non-graminoid proportion from GLM.

#### Relationship between dietary variation and variation in MS

To test the hypothesis that a species (or a population) with greater dietary variation shows greater mesowear variation, we performed statistical tests for both interspecific and intraspecific data. We used standard deviation of MS as a proxy of mesowear variation within a species (or a population) with consideration of its statistical characteristics. First, standard deviation is affected by sample size; i.e., it becomes smaller with increasing sample size. However, we did not found a negative relationship between standard deviation of MS and sample size in both interspecific and intraspecific data sets (*P*>0.10 for either case). Next, the mean and standard deviation of MS were significantly correlated; i.e., a species (or a population) with greater MS shows greater deviation in MS, irrespective of its dietary variation (for interspecific data, N = 68, *r* = 0.30, *P* = 0.01; for intraspecific data, N = 15, *r* = 0.79, *P* = 0.0005). Thus, we adjusted the standard deviation by regressing it against the MS mean and obtaining residuals from the regression line, separately for interspecific and intraspecific data sets. We did not use coefficient of variance (CV, the standard deviation divided by the mean), because the means of MS took zero values in some species. For interspecific data, we compared the adjusted standard deviation of MS between species with greater dietary variation (“variable”  =  yes) and those with lesser variation (“variable”  =  no). For intraspecific data of the sika deer populations, we tested the correlation between the variability of graminoid consumption and the adjusted standard deviation of MS.

#### Intraspecific correlations among dental variables

We tested correlations among the M_3_ hypsodonty index, molar wear rate, and MS of the sika deer. Because data for the M_3_ hypsodonty index and molar wear rate were limited to female samples, the statistical tests were additionally performed using MS of female subsamples alone. All statistical analyses were performed using JMP release 10.0.2 (SAS Institute Inc., Cary, North Carolina).

## Results

### Relationship between dental variables and ecological variables

**Figure 3:**
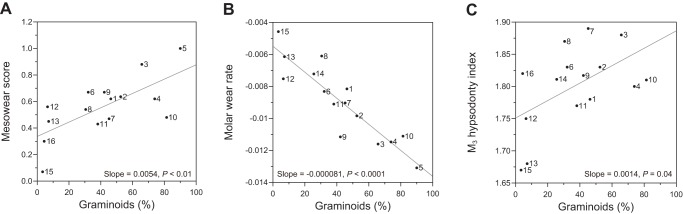
Relationship between dental variables (A: mesowear score, B: molar wear rate, and C: M_3_ hypsodonty index) and graminoid proportion in extant sika deer. Population numbers correspond to those in Tables 1 and 2. The linear regression line is drawn for each plot, along with the regression slope and its *P*-value.

MS, the molar wear rate, and the M_3_ hypsodonty index of each sika deer population are presented in Table 2, along with the sample size used to obtain these data. We found significant differences of MS among sika deer populations (the Kruskal-Wallis test, d.f.  = 14, *X* ^2^ = 83.75, *P*<0.0001). Using population mean of MS (the response variable) and dietary and environmental variables (the predictor variables), we conducted GLM with stepwise model selection. As a result, the model including only graminoid proportion was selected (Table S3, N = 15, slope  = 0.0054, *P*<0.01, Fig. 3A), that is, proportion of graminoid in diet was the most influential factor determining MS. Similarly, we conducted stepwise model selection for the molar wear rate and the M_3_ hypsodonty index (Table S3) and selected the model with graminoid proportion alone as the best model for either case (for the molar wear rate, N = 15, slope  = −0.000081, *P*<0.0001, Fig. 3B; for the M_3_ hypsodonty index, N = 15, slope  = 0.0014, *P* = 0.04, Fig. 3C).

**Figure 4:**
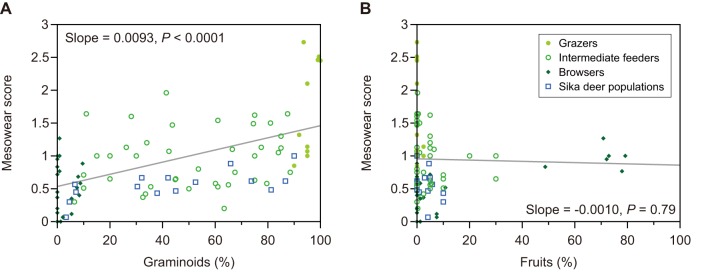
Relationship between the mesowear score and (A) graminoid proportion in diet and (B) fruit proportion in diet among 75 living species of ungulate. Three dietary types are defined: grazers consuming ≥90% graminoids, browsers consuming <10% graminoids, and intermediate feeders. The keys for both plots are in plot (B). Data from 15 populations of extant sika deer are overlaid but were not used for regression analyses. The linear regression line using 75 species is drawn for each plot, along with the regression slope and its *P*-value.

The synthesized data for diet and MS of living ungulates are provided in Table S2. Similar to the case of intraspecific relationship, we found that MS was significantly affected by graminoid proportion but not fruit proportion (for graminoids, N = 73, slope  = 0.0093, *P*<0.0001, Fig. 4A; for fruits, N = 73, slope  = −0.00096, *P* = 0.79, Fig. 4B), concordant with the results of the study by Kaiser et al. [40]. Even when we limited the data to selenodont species with number of individuals more than 10, the relationship between graminoid proportion and MS was still significant (for graminoids, N = 47, slope  = 0.0073, *P*<0.001; for fruits, N = 47, slope  = 0.00066, *P* = 0.85). The regression slopes for graminoids – MS relationship were not significantly different between the two data sets (95% confidence interval of slope, for 73 ungulate species, 0.0060 to 0.0125; for 47 selenodont species with N>10, 0.0035 to 0.0112), concordant with the fact that MS was less affected by phylogenetic relationship among species [40].

### Relationship between dietary variation and MS variation

From analysis of interspecific data (N = 68), the relationship between MS and the MS standard deviation was described by the following regression equation:
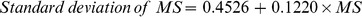
(1)


**Figure 5:**
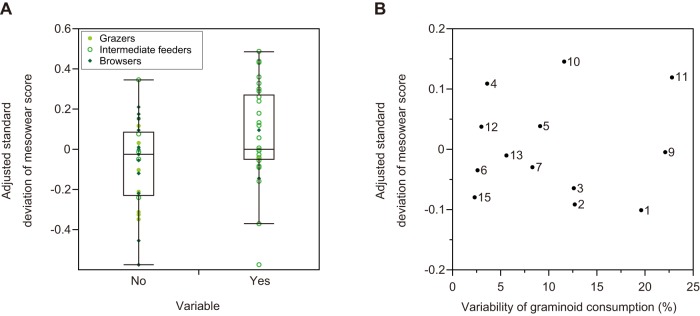
Relationship between diet variability and variation in mesowear score (MS). (A) The interspecific comparison between groups with variable (“yes”) and non-variable (“no”) diets. The three dietary types are the same as those in Fig. 4. The box encloses the 25^th^ and 75^th^ percentiles, with the horizontal line representing the median (50^th^ percentile). The whiskers (the perpendicular lines stretching from the top and bottom of the box) show the range of observed values that fall within 1.5× interquartile range (height of the box). (B) The intraspecific relationship between seasonal variability in graminoid consumption and MS variation among 13 sika deer populations. Population numbers correspond to those in Tables 1 and 2.

The deviation from the regression line was defined as the adjusted standard deviation of MS. It was shown that species with greater dietary variation (“variable”  =  yes) had significantly higher positive values of the adjusted standard deviation of MS than those with lesser dietary variation (“variable”  =  no) (Student's *t*-test, *t* = 2.43, d.f.  = 57, *P* = 0.02, Fig. 5A). The 95% confidence interval for the adjusted standard deviation of MS was −0.162 to 0.008 for species with lesser dietary variation and −0.016 to 0.152 for those with greater dietary variation. In contrast to the interspecific results, no significant correlation was observed between the variability of graminoid consumption and the adjusted standard deviation of MS among the sika deer populations (N = 13, *r* = 0.04, *P* = 0.90, Fig. 5B).

### Intraspecific correlations among dental variables

**Figure 6:**
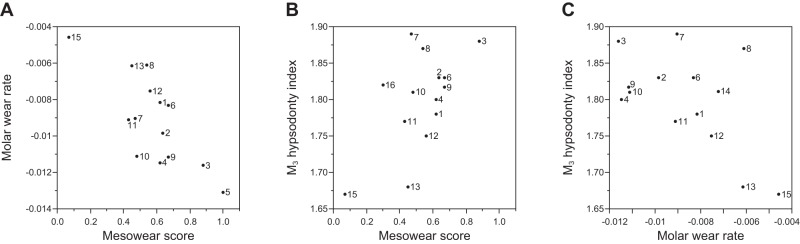
Correlations among dental variables (mesowear score, molar wear rate, and M_3_ hypsodonty index) in sika deer. Population numbers correspond to those in Tables 1 and 2.

As expected from our assumptions on the process of dental wear, we found significant correlations among dental variables. The molar wear rate and MS were negatively correlated (N = 14, *r* = −0.76, *P* = 0.002, Fig. 6A; for female only subsample for MS, N = 14, *r* = −0.70, *P* = 0.01), indicating that deer from habitats that accelerate the molar wear rate have a high MS ( =  more rounded molar cusp). MS and the M_3_ hypsodonty index were positively correlated (N = 14, *r* = 0.59, *P* = 0.03, Fig. 6B; for female only subsample for MS, N = 14, *r* = 0.64, *P* = 0.01). Finally, we found a significant correlation between the molar wear rate and the M_3_ hypsodonty index (both variables obtained only from females, N = 14, *r* = −0.55, *P* = 0.04, Fig. 6C), indicating more rapid molar wear is associated with more hypsodont M_3_.

## Discussion

### Quantitative evaluation of mesowear analysis using sika deer samples

Statistical tests revealed that MS was significantly correlated with graminoid proportion but not with annual precipitation, confirming the reported interspecific trend [40]–[42]. Because interspecific comparisons are affected by shared ancestry, Kaiser et al. [40] adopted phylogenetically controlled statistics. However, molar morphology is more uniform among populations within a species. Therefore, our results clearly indicate that graminoid consumption affects mesowear, further supporting the use of mesowear analysis for dietary reconstruction.

We also quantitatively tested whether dietary variation relates to mesowear variation, both within and among species. As expected, species with greater dietary variation showed greater MS variation than those with lesser dietary variation. The two groups overlap, but the 95% confidence interval indicated that the negative value of the adjusted standard deviation of MS strongly implies that the species exhibit lesser dietary variation. Therefore, the adjusted standard deviation of MS, which is obtained from formula (1), can be used as a proxy for dietary variation in extinct species. However, we did not find a significant correlation between the variability of graminoid consumption and MS variation among the sika deer populations. It is possible that compared with species that inhabit environments with drastic seasonal change (e.g., the rainy/dry season) and clearly switch their diets, the Japanese sika deer inhabit moderate environments with less fluctuation, and thus, their diet variation may be much lesser.

From intraspecific comparisons of the sika deer populations, we found a significant correlation between MS and the molar wear rate, i.e., deer with more rounded molar cusps also showed more rapid molar wear. This finding is also important in that mesowear has the potential to indicate a temporal change in the molar wear rate, which normally cannot be reconstructed for fossil samples, because of the lack of age data for individual animals. For example, if we have two fossil assemblages of the same species from different geological ages and find a significant difference in MS between them, then we can infer that the species may have altered their diet, resulting in acceleration or deceleration of the molar wear rate.

### Factors influencing molar wear: intrinsic silica or external grit

In addition to the assessment of the utility of mesowear analysis for dietary reconstruction, we evaluated the ecological factors influencing molar wear. The correlation between graminoid proportion and the molar wear rate, which was presented in the study by Ozaki et al. [45] but was not statistically significant, was significant in the present study that included additional populations. This implies that graminoid proportion is the most influential factor determining the molar wear rate. Annual precipitation in habitat was not correlated with the molar wear rate. This result contradicts the supposition made in the study by Kaiser et al. [40] that external abrasives influence the tooth wear rate without affecting cusp morphology, which they inferred from the negative correlation between annual precipitation and the hypsodonty index among ungulates. Comparative data on the molar wear rates in other ungulate species are limited, but Solounias et al. [53] provided the molar wear rates of nine ruminant species. From a close investigation of their data combined with additional data on graminoid proportion (this study) and annual precipitation in habitat [40], we found that the molar wear rate was significantly correlated with graminoid proportion but not with annual precipitation in habitat (graminoids, N = 9, *r* = 0.70, *P* = 0.03; annual precipitation, N = 9, *r* = −0.04, *P* = 0.92; see Table S4 and Figure S1 for detailed analysis). Although more precise data on the molar wear rates of other species are needed, the combined results of intraspecific and interspecific comparisons implied that annual precipitation in habitat does not influence the tooth wear rate. Therefore, it is possible that annual precipitation itself does not considerably influence the amount of external abrasives adhered to the plant surface. Rather, annual precipitation has a strong impact on vegetation; plants growing in open habitats tend to be more contaminated with dust or grit than those growing in closed habitats. Figure 4A supports this view, which clearly shows higher values (up to 1.5) of MS of intermediate feeders (all are open–intermediate habitat dwellers) than the forest-dwelling sika deer populations with similar graminoid consumption. We infer that the globally observed pattern of increasing hypsodonty in ungulates concomitant with increasing annual precipitation (or increasing openness in habitats) [7], [33], [40] is caused by the ingestion of both internal and external abrasives in open habitats, although the effect of the former is sometimes obscured in the presence of the strong impact of the latter.

There are two possible ways for animals to ingest the external abrasives; they subsidiary ingest those adhered to the plant surface and/or they directly ingest soil or sand due to feeding at the ground level. Damuth and Janis [7] discussed these possibilities in detail, insisting that soil ingestion due to ground level feeding has a strong impact on the tooth wear rate, regardless of actual grass consumption of ungulates in open habitats. Could the soil ingestion explanation apply to sika deer? If so, the correlation between graminoid proportion and the molar wear rate is merely a by-product of feeding near the ground and ingesting more soil when deer crop grass. This possibility, however, is denied when we closely examine the graminoid species eaten by deer. It has been shown that sika deer in northern areas rely on dwarf bamboo, which is evergreen and abundant in the forest understory [44]. The physical structure of dwarf bamboo is unique in that it has long stems and leaves grow at the tops of the stems. Because of these characteristics, deer do not feed near the ground when feeding on dwarf bamboo; in most cases, the height of dwarf bamboo is 40–60 cm. Among the 11 sika deer populations which habitually consume graminoids (graminoid proportion exceeds 10% of the annual diet), six populations (population Nos. 1, 2, 4, 6, 8, and 9) consume a higher proportion of dwarf bamboo. Other shorter graminoids, such as sedges (genus *Carex*) with heights up to 30 cm, are found more often in the diets of deer in southern areas (Nos. 7, 11, and 14). The shortest graminoid is *Zoysia japonica* (height, ≤10 cm), a lawn-like grass that can tolerate high grazing pressure and is predominantly found in areas with high deer density (Kinkazan Island, No. 3 and Nara Park, No. 10). If soil ingestion due to feeding at the ground level has a strong impact on the molar wear rate, then we would expect that deer feeding on dwarf bamboo would show a slower wear rate than deer feeding on sedges or lawn-like grass, when the overall graminoid proportion is similar. From Fig. 3B, the six populations feeding high proportions of dwarf bamboo are not above the regression line, indicating that molar wear in these deer are not slower than those in the deer feeding on shorter graminoids. Two populations that rely on lawn-like grass (Nos. 3 and 10) do show more rapid molar wear. However, the Nikko population (No. 4), which feeds on a similar graminoid proportion but predominantly consumes dwarf bamboo, has a similar molar wear rate. Deer from the Ashio population show the most rapid molar wear among the 15 populations. Precise data on the graminoid species eaten by the Ashio deer were not available, but Chinese silver grass (*Miscanthus sinensis*), which grows to 0.8–2 m tall, made a large contribution during winter [54]. Therefore, the correlation between graminoid proportion and the molar wear rate is not merely the by-product of feeding near the ground. Among these graminoid species eaten by sika deer, silica content also varies from 1% (sedges) to 5% (dwarf bamboo) per dry matter. Though we did not measure the silica content in diet for each sika deer population, we can estimate it from published data on silica content of graminoid species [5]. We found that both MS and the molar wear rate were significantly correlated with the estimated silica content (%) in deer diet (for MS, N = 14, slope  = 0.1487, P = 0.03; for the molar wear rate, N = 14, slope  = −0.0070, *P*<0.01; see Table S5 and Figure S2 for detailed analysis). Therefore, our empirical data clearly showed that grass phytolith plays a significant role in abrasion, necessitating to reconsider the results of the experimental studies [9], [12].

### Evolutionary consequences of the accelerated molar wear rate and evolution of hypsodonty within a species

The main factor influencing the molar wear rate (and MS), at least in extant sika deer, would thus be the proportion of graminoids consumed. Then, what is the evolutionary outcome of the accelerated molar wear rate? Because Ozaki et al. [45] showed a significant positive correlation between life expectancy and molar durability, which was defined as molar height divided by the molar wear rate, greater molar durability ( = higher molars) is adaptive, and higher molars should be selected in environments that promote tooth wear. Following this expectation, we observed a trend of increase in the M_3_ hypsodonty index with an acceleration in the molar wear rate not only among the nine ruminant species (N = 9, slope  = 0.7424, *P* = 0.07, see Table S4 and Figure S1 for a detailed analysis) but also among sika deer populations (Fig. 6C). The intraspecific correlation appears to imply the evolution of more hypsodont molars against rapid tooth wear. However, we have some reservations regarding this conclusion because the degree of hypsodonty differs significantly between two phylogenetically distinct lineages of sika deer [48], namely the northern and southern lineages, which diverged approximately 0.3–0.5 Mya [55], [56]. In Fig. 6C, populations of the northern lineage (population Nos. 1–10) tend to have more hypsodont molars than populations of the southern lineage (Nos. 11–15). The hypsodonty index increased with an accelerating molar wear rate in the southern lineage (N = 5, *r* = −0.74, *P* = 0.14) but not in the northern lineage (N = 9, *r* = 0.19, *P* = 0.62). We present the following hypotheses: (1) the ancestral population of the northern lineage acquired more hypsodont molars in the past, possibly under a strong environmental pressure, which promoted molar wear, (2) after subdivision into local populations, molar height has not changed measurably because of relaxed selection and/or insufficient duration of selection, especially for populations of the northern lineage. Genetic evidence indicated that the greatest genetic differentiation among populations was found in the Kyushu area (southern lineage) among regional groups in Japan [57], and that among the Honshu (the northern lineage) sika deer population, differentiation occurred more recently because of habitat fragmentation [58]. These findings appear to support the hypothesis that populations of the northern lineage require additional time for measurable differentiation of molar hypsodonty, given their current molar wear rate. This hypothesis could be tested by the examination of ancestral conditions and chronological changes in molar hypsodonty in sika deer using samples from paleontological or archaeological sites.

## Conclusions

In reference to the evolutionary trend of increasing cheek tooth height in herbivorous ungulates, the causes of dental abrasion have long been debated. Interspecific comparisons of extant ungulates have revealed that both phytoliths in grass and external abrasive matter may play equally important roles (although recent discussions give the latter a more prominent role). From analysis of extant sika deer inhabiting various environments and consuming graminoids in varying proportions, notably the northern sika deer “forest grazers,” we quantitatively evaluated the effect of graminoid consumption on MS, the molar wear rate, and the M_3_ hypsodonty index. We found that graminoid proportion in diets alone was correlated with MS and the molar wear rate, implying that phytoliths in grass can abrade dental tissues, at least in Japanese sika deer. Some grazing species inhabiting drier and more open habitats may ingest a considerable amount of external abrasives, which will mask the effect of phytoliths as abrading agents. Nevertheless, even in the closed forest habitat that is seen in the current range of sika deer, grass has a prominent role in dental abrasion and therefore can be directly responsible for evolution of hypsodonty. In other words, we do not need to assume the existence of extensive grassland habitats in past when evolution of hypsodonty would begin to emerge [11], [59], [60]. In extant sika deer, the evolutionary acquisition of more hypsodont molars corresponding to a higher molar wear rate may be occurring, specifically for populations of the southern lineage. However, sika deer populations have diverged relatively recently compared with the divergence of living ungulate species. Thus, inadequate duration of selection may be responsible for the absence of noticeable changes in molar hypsodonty among the sika deer populations. The divergence time between the two phylogenetic lineages of sika deer (c.a. 0.3–0.5 Mya) [55], [56], which showed measurable differentiation in molar hypsodonty, may provide the minimum estimate of time required for evolution of hypsodonty.

Our analyses lend further support for the use of mesowear analysis as a paleodiet estimation method. We present the novel finding that dietary information is revealed not only by the MS mean, which correlates with the proportion of graminoids consumed, but also by the MS standard deviation, which is associated with seasonal/regional variation in diet. Dietary variation in extinct species has seldom been investigated, because (1) although seasonal variation in diet of intermediate feeders can be resolved by microwear analysis of a large number of samples [18], [30], [61], it is time consuming and suffered by high inter- and intra-observer errors [62], and (2) although sequential sampling along the vertical axis of molars in stable isotopic analysis can also resolve seasonal variation in diet [63], it is also time consuming and can only reveal variation during molar formation, which may occur over a short period in brachydont species. Investigation of the MS standard deviation can provide a first inference on whether the species in question shows dietary variation, without additional effort. It also potentially offers a way to investigate chronological change in dietary niche breadth for fossil species. Further quantitative evaluation of the MS standard deviation, for example, by correlation tests between the standard deviation of MS and quantitative measurements of dietary variation or stable isotopic values, will further elucidate the utility of the MS standard deviation.

## Supporting Information

Figure S1 Scatter plots showing relationships between (A) graminoid proportion in diet and the molar wear rate, (B) annual precipitation in habitat and the molar wear rate, and (C) the molar wear rate and the M_3_ hypsodonty index among nine ruminants. Data are presented in Table S4. The linear regression line is drawn for each plot, along with the regression slope and its *P*-value.
(JPG)

Figure S2 Scatter plots showing relationships between estimated silica content in diet and (A) the molar wear rate, (B) the mesowear score among sika deer populations. Data are presented in Table S5. The linear regression line is drawn for each plot, along with the regression slope and its *P*-value.
(JPG)

Table S1 Museum and specimen information of sika deer, along with original mesowear data of individual specimens.
(DOC)

Table S2 Synthesized data on mesowear score (MS) and dietary information of 75 ungulate species.
(DOC)

Table S3 Selection of the best model predicting MS, the molar wear rate or the M_3_ hypsodonty index of the Japanese sika deer. The best models with the lowest AICc value are in bold font. In the case where the M_3_ hypsodonty index was the response variable, the model with the lowest AICc value included both graminoid proportion and annual precipitation. However, annual precipitation was not statistically significant (*P* = 0.06). Therefore, we selected the model included only graminoid proportion as the best model for the M_3_ hypsodonty index. The predictor variables are: G =  graminoid proportion; F =  fruit proportion; AP =  annual precipitation; G×AP  =  interaction between graminoid proportion and annual precipitation; F×AP =  interaction between fruit proportion and annual precipitation. K =  number of parameters in the model; AICc  =  Akaike information criterion corrected for sample size; + =  factor included in the model.
(DOC)

Table S4 Comparative data on the molar wear rate, the M_3_ hypsodonty index, and ecological variables for nine living ruminants.
(DOC)

Table S5 Estimation of silica content in deer diet. Estimated silica content (%) is further used for regression analyses testing the relationship between silica content and dental variables (See Fig. S2).
(DOC)
