# Supplementary figures and images for: The Inter-Relationship between Dietary and Environmental Properties and Tooth Wear: Comparisons of Mesowear, Molar Wear Rate, and Hypsodonty Index of Extant Sika Deer Populations
Source: PLoS One. 2014 Mar 6;9(3):e90745. doi: 10.1371/journal.pone.0090745 (PMC3946258; doi:10.1371/journal.pone.0090745)

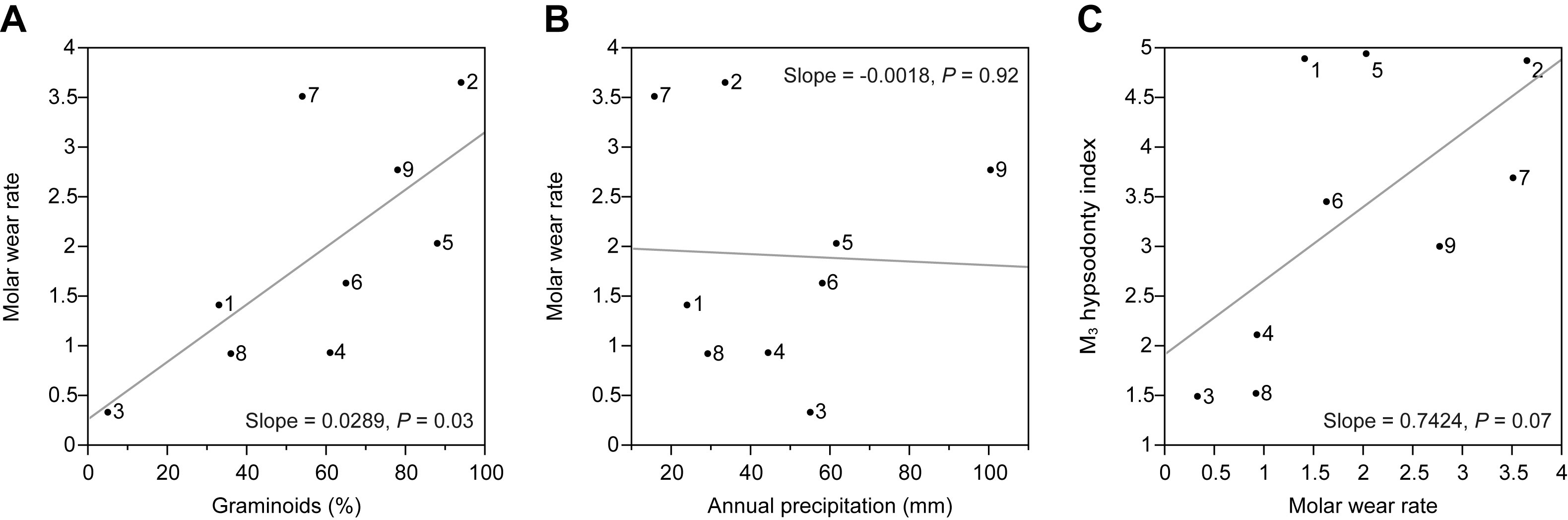

Supplement: Figure S1 — Scatter plots showing relationships between (A) graminoid proportion in diet and the molar wear rate, (B) annual precipitation in habitat and the molar wear rate, and (C) the molar wear rate and the M3 hypsodonty index among nine ruminants. Data are presented in Table S4. The linear regression line is drawn for each plot, along with the regression slope and its P-value. (JPG) [file pone.0090745.s001.jpg]

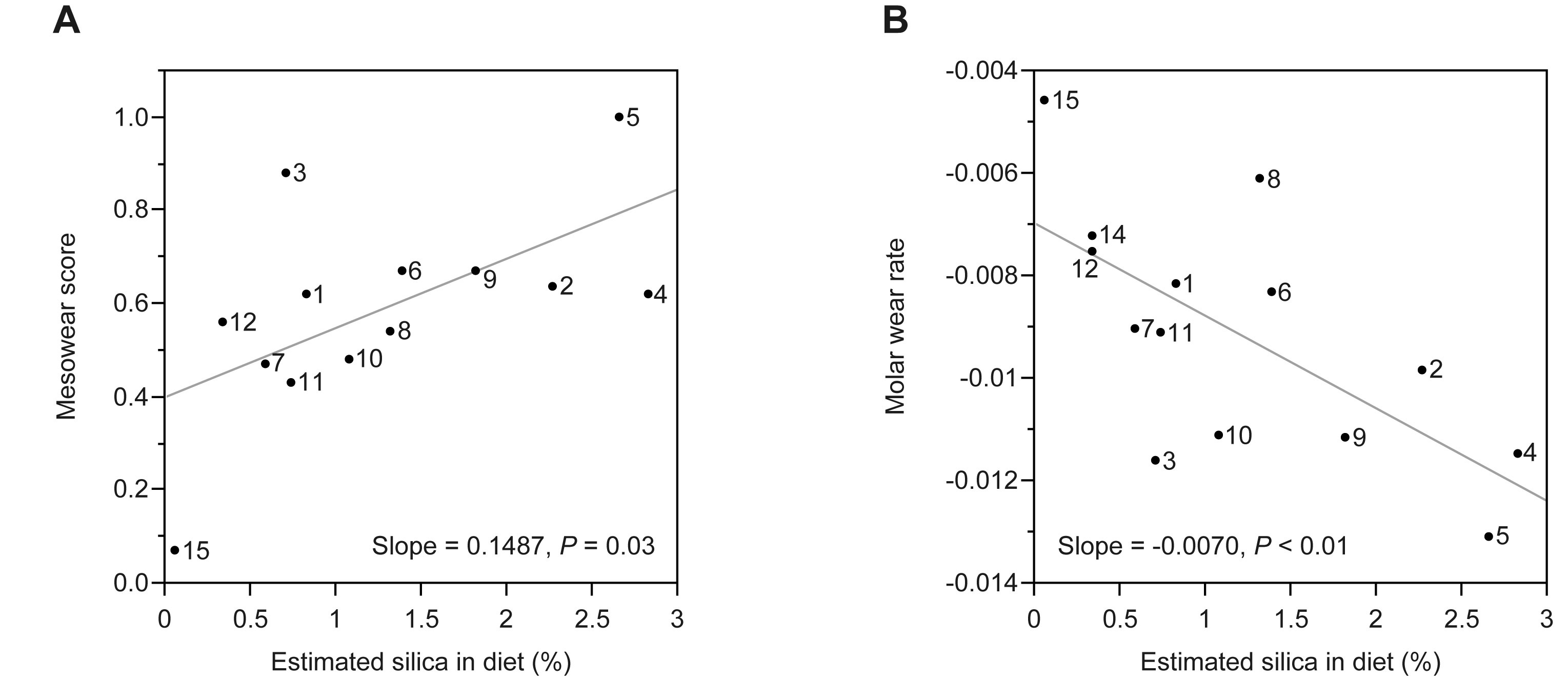

Supplement: Figure S2 — Scatter plots showing relationships between estimated silica content in diet and (A) the molar wear rate, (B) the mesowear score among sika deer populations. Data are presented in Table S5. The linear regression line is drawn for each plot, along with the regression slope and its P-value. (JPG) [file pone.0090745.s002.jpg]
