# Supplementary material for: The Inter-Relationship between Dietary and Environmental Properties and Tooth Wear: Comparisons of Mesowear, Molar Wear Rate, and Hypsodonty Index of Extant Sika Deer Populations
Source: PLoS One. 2014 Mar 6;9(3):e90745. doi: 10.1371/journal.pone.0090745 (PMC3946258; doi:10.1371/journal.pone.0090745)
Supplement: Table S1 — Museum and specimen information of sika deer, along with original mesowear data of individual specimens. (DOC) [file pone.0090745.s003.doc]

Supplementary Table S1. Museum and specimen information of sika deer, along with original mesowear data of individual specimens.

| Pop No. | Locality | Specimen ID | Sex | Side | low | high | sharp | round | blunt | Mesowear score^*^ | Collection^**^ |
| --- | --- | --- | --- | --- | --- | --- | --- | --- | --- | --- | --- |
| 1 | Eastern Hokkaido | HKD-0401 | F | L | 0 | 1 | 0 | 1 | 0 | 1 | UMUT |
| 1 | Eastern Hokkaido | HKD-0409 | F | L | 0 | 1 | 1 | 0 | 0 | 0 | UMUT |
| 1 | Eastern Hokkaido | HKD-0413 | F | L | 0 | 1 | 1 | 0 | 0 | 0 | UMUT |
| 1 | Eastern Hokkaido | HKD-0431 | F | L | 0 | 1 | 0 | 1 | 0 | 1 | UMUT |
| 1 | Eastern Hokkaido | HKD-0440 | M | L | 0 | 1 | 0 | 1 | 0 | 1 | UMUT |
| 1 | Eastern Hokkaido | HKD-0444 | M | L | 0 | 1 | 0 | 1 | 0 | 1 | UMUT |
| 1 | Eastern Hokkaido | HKD-0449 | M | L | 0 | 1 | 0 | 1 | 0 | 1 | UMUT |
| 1 | Eastern Hokkaido | HKD-0457 | M | L | 0 | 1 | 0 | 1 | 0 | 1 | UMUT |
| 1 | Eastern Hokkaido | HKD-0460 | M | L | 0 | 1 | 0 | 1 | 0 | 1 | UMUT |
| 1 | Eastern Hokkaido | M0211 | F | R | 0 | 1 | 0 | 1 | 0 | 1 | TPM |
| 1 | Eastern Hokkaido | M0213 | F | L | 0 | 1 | 0 | 1 | 0 | 1 | TPM |
| 1 | Eastern Hokkaido | M0214 | F | L | 0 | 1 | 1 | 0 | 0 | 0 | TPM |
| 1 | Eastern Hokkaido | M0215 | F | L | 0 | 1 | 0 | 1 | 0 | 1 | TPM |
| 1 | Eastern Hokkaido | M0216 | M | L | 0 | 1 | 1 | 0 | 0 | 0 | TPM |
| 1 | Eastern Hokkaido | M0217 | F | L | 0 | 1 | 0 | 1 | 0 | 1 | TPM |
| 1 | Eastern Hokkaido | M222 | M | L | 0 | 1 | 1 | 0 | 0 | 0 | TPM |
| 1 | Eastern Hokkaido | M226 | M | L | 0 | 1 | 1 | 0 | 0 | 0 | TPM |
| 1 | Eastern Hokkaido | M227 | M | L | 0 | 1 | 0 | 1 | 0 | 1 | TPM |
| 1 | Eastern Hokkaido | M228 | M | L | 0 | 1 | 0 | 1 | 0 | 1 | TPM |
| 1 | Eastern Hokkaido | M229 | F | L | 0 | 1 | 1 | 0 | 0 | 0 | TPM |
| 1 | Eastern Hokkaido | M230 | M | L | 0 | 1 | 1 | 0 | 0 | 0 | TPM |
| 2 | Mt. Goyo | IT-84101 | F | L | 0 | 1 | 0 | 1 | 0 | 1 | UMUT |
| 2 | Mt. Goyo | IT-8846 | F | L | 0 | 1 | 0 | 1 | 0 | 1 | UMUT |
| 2 | Mt. Goyo | IT-88101 | F | L | 0 | 1 | 0 | 1 | 0 | 0 | UMUT |
| 2 | Mt. Goyo | IT-90167 | F | L | 0 | 1 | 1 | 0 | 0 | 0 | UMUT |
| 2 | Mt. Goyo | IT-92127 | F | L | 0 | 1 | 0 | 1 | 0 | 1 | UMUT |
| 2 | Mt. Goyo | IT-92499 | M | L | 0 | 1 | 0 | 1 | 0 | 1 | UMUT |
| 2 | Mt. Goyo | IT-92505 | F | L | 0 | 1 | 0 | 1 | 0 | 1 | UMUT |
| 2 | Mt. Goyo | IT-97620 | M | L | 0 | 1 | 1 | 0 | 0 | 0 | UMUT |
| 2 | Mt. Goyo | IT-97639 | M | L | 0 | 1 | 1 | 0 | 0 | 0 | UMUT |
| 2 | Mt. Goyo | IT-987 | M | L | 0 | 1 | 0 | 1 | 0 | 1 | UMUT |
| 2 | Mt. Goyo | IT00dead3 | F | L | 0 | 1 | 1 | 0 | 0 | 0 | UMUT |
| 2 | Mt. Goyo | IT0008 | F | L | 0 | 1 | 0 | 1 | 0 | 1 | UMUT |
| 2 | Mt. Goyo | 20090526 | F | L | 0 | 1 | 0 | 1 | 0 | 1 | UMUT |
| 2 | Mt. Goyo | IT-0062 | M | R | 0 | 1 | 0 | 1 | 0 | 1 | UMUT |
| 2 | Mt. Goyo | IT-01467 | M | L | 0 | 1 | 0 | 1 | 0 | 1 | UMUT |
| 2 | Mt. Goyo | IT-0229 | M | L | 0 | 1 | 1 | 0 | 0 | 0 | UMUT |
| 2 | Mt. Goyo | IT-02114 | M | L | 0 | 1 | 0 | 1 | 0 | 1 | UMUT |
| 2 | Mt. Goyo | IT-02162 | M | L | 0 | 1 | 0 | 1 | 0 | 1 | UMUT |
| 2 | Mt. Goyo | IT-02163 | M | L | 0 | 1 | 1 | 0 | 0 | 0 | UMUT |
| 2 | Mt. Goyo | IT-0510 | M | L | 0 | 1 | 1 | 0 | 0 | 0 | UMUT |
| 2 | Mt. Goyo | NOID1981/12 | F | L | 0 | 1 | 0 | 1 | 0 | 1 | UMUT |
| 2 | Mt. Goyo | NOID1984/7/8 | F | L | 0 | 1 | 0 | 1 | 0 | 1 | UMUT |
| 3 | Kinkazan Island | 84153 | F | L | 0 | 1 | 0 | 1 | 0 | 1 | UMUT |
| 3 | Kinkazan Island | 84174 | F | L | 0 | 1 | 0 | 1 | 0 | 1 | UMUT |
| 3 | Kinkazan Island | 84181 | F | L | 0 | 1 | 1 | 0 | 0 | 0 | UMUT |
| 3 | Kinkazan Island | 84185 | F | L | 0 | 1 | 0 | 1 | 0 | 1 | UMUT |
| 3 | Kinkazan Island | 84191 | F | L | 0 | 1 | 0 | 1 | 0 | 1 | UMUT |
| 3 | Kinkazan Island | 84192 | F | L | 0 | 1 | 1 | 0 | 0 | 0 | UMUT |
| 3 | Kinkazan Island | 84195 | F | L | 0 | 1 | 0 | 1 | 0 | 1 | UMUT |
| 3 | Kinkazan Island | (84)266 | F | L | 0 | 1 | 0 | 1 | 0 | 1 | UMUT |
| 3 | Kinkazan Island | (84)3 | F | L | 0 | 1 | 1 | 0 | 0 | 0 | UMUT |
| 3 | Kinkazan Island | 84222 | F | L | 0 | 1 | 0 | 1 | 0 | 1 | UMUT |
| 3 | Kinkazan Island | (84)284 | F | L | 0 | 1 | 0 | 1 | 0 | 1 | UMUT |
| 3 | Kinkazan Island | MG95078 | M | L | 0 | 1 | 0 | 1 | 0 | 1 | UMUT |
| 3 | Kinkazan Island | MG95080 | M | L | 1 | 0 | 0 | 1 | 0 | 2 | UMUT |
| 3 | Kinkazan Island | MGK96018 | M | L | 0 | 1 | 0 | 1 | 0 | 1 | UMUT |
| 3 | Kinkazan Island | MGK96037 | F | L | 0 | 1 | 0 | 1 | 0 | 1 | UMUT |
| 3 | Kinkazan Island | MGK96044 | M | L | 0 | 1 | 0 | 1 | 0 | 1 | UMUT |
| 3 | Kinkazan Island | MGK97085 | M | L | 0 | 1 | 0 | 1 | 0 | 1 | UMUT |
| 3 | Kinkazan Island | MGK97086 | F | L | 1 | 0 | 0 | 1 | 0 | 2 | UMUT |
| 3 | Kinkazan Island | MGK97087 | M | L | 1 | 0 | 0 | 1 | 0 | 2 | UMUT |
| 3 | Kinkazan Island | MGK97093 | F | L | 0 | 1 | 0 | 1 | 0 | 1 | UMUT |
| 3 | Kinkazan Island | MGK97096 | M | L | 1 | 0 | 0 | 1 | 0 | 2 | UMUT |
| 3 | Kinkazan Island | MGK97121 | F | L | 0 | 1 | 0 | 1 | 0 | 1 | UMUT |
| 3 | Kinkazan Island | MGK97123 | F | L | 0 | 1 | 1 | 0 | 0 | 0 | UMUT |
| 3 | Kinkazan Island | MGK97131 | F | L | 1 | 0 | 1 | 0 | 0 | 2.5 | UMUT |
| 3 | Kinkazan Island | MGK97132 | F | L | 0 | 1 | 1 | 0 | 0 | 0 | UMUT |
| 3 | Kinkazan Island | MGK97133 | F | L | 0 | 1 | 0 | 1 | 0 | 1 | UMUT |
| 3 | Kinkazan Island | MGK97136 | M | L | 0 | 1 | 1 | 0 | 0 | 0 | UMUT |
| 3 | Kinkazan Island | MGK97138 | F | L | 0 | 1 | 0 | 1 | 0 | 1 | UMUT |
| 3 | Kinkazan Island | MGK97139 | F | L | 0 | 1 | 1 | 0 | 0 | 0 | UMUT |
| 3 | Kinkazan Island | MGK97144 | M | L | 0 | 1 | 0 | 1 | 0 | 1 | UMUT |
| 3 | Kinkazan Island | MGK97146 | M | L | 0 | 1 | 0 | 1 | 0 | 1 | UMUT |
| 3 | Kinkazan Island | MGK97147 | M | L | 1 | 0 | 0 | 1 | 0 | 2 | UMUT |
| 3 | Kinkazan Island | MGK97148 | F | L | 0 | 1 | 1 | 0 | 0 | 0 | UMUT |
| 3 | Kinkazan Island | MGK97149 | M | L | 1 | 0 | 1 | 0 | 0 | 2.5 | UMUT |
| 3 | Kinkazan Island | MGK97151 | F | L | 0 | 1 | 1 | 0 | 0 | 0 | UMUT |
| 3 | Kinkazan Island | MGK97158 | F | L | 0 | 1 | 1 | 0 | 0 | 0 | UMUT |
| 3 | Kinkazan Island | MGK97160 | F | L | 1 | 0 | 0 | 1 | 0 | 2 | UMUT |
| 3 | Kinkazan Island | MGK97162 | M | L | 1 | 0 | 0 | 1 | 0 | 2 | UMUT |
| 3 | Kinkazan Island | MGK97214 | F | L | 0 | 1 | 0 | 1 | 0 | 1 | UMUT |
| 3 | Kinkazan Island | MGK97216 | M | L | 0 | 1 | 1 | 0 | 0 | 0 | UMUT |
| 3 | Kinkazan Island | MGK97218 | F | L | 0 | 1 | 0 | 1 | 0 | 1 | UMUT |
| 3 | Kinkazan Island | MGK97235 | M | L | 0 | 1 | 0 | 1 | 0 | 1 | UMUT |
| 3 | Kinkazan Island | MGK97239 | F | L | 1 | 0 | 0 | 1 | 0 | 2 | UMUT |
| 3 | Kinkazan Island | MGK97240 | M | L | 0 | 1 | 1 | 0 | 0 | 0 | UMUT |
| 3 | Kinkazan Island | MGK97258 | M | L | 0 | 1 | 0 | 1 | 0 | 1 | UMUT |
| 3 | Kinkazan Island | MGK97260 | F | L | 0 | 1 | 0 | 1 | 0 | 1 | UMUT |
| 3 | Kinkazan Island | MGK97263 | M | L | 0 | 1 | 0 | 1 | 0 | 1 | UMUT |
| 3 | Kinkazan Island | MGK97265 | F | L | 0 | 1 | 0 | 1 | 0 | 1 | UMUT |
| 3 | Kinkazan Island | MGK97267 | F | L | 0 | 1 | 1 | 0 | 0 | 0 | UMUT |
| 3 | Kinkazan Island | MGK97268 | M | L | 1 | 0 | 0 | 1 | 0 | 2 | UMUT |
| 3 | Kinkazan Island | MGK97269 | F | L | 0 | 1 | 1 | 0 | 0 | 0 | UMUT |
| 3 | Kinkazan Island | MGK97270 | M | L | 0 | 1 | 0 | 1 | 0 | 1 | UMUT |
| 3 | Kinkazan Island | MGK97271 | M | L | 1 | 0 | 0 | 1 | 0 | 2 | UMUT |
| 3 | Kinkazan Island | MGK97272 | F | L | 0 | 1 | 1 | 0 | 0 | 1 | UMUT |
| 3 | Kinkazan Island | MGK97273 | F | L | 0 | 1 | 1 | 0 | 0 | 1 | UMUT |
| 3 | Kinkazan Island | MGK97274 | F | L | 0 | 1 | 0 | 1 | 0 | 1 | UMUT |
| 3 | Kinkazan Island | MG97287 | M | L | 0 | 1 | 1 | 0 | 0 | 0 | UMUT |
| 3 | Kinkazan Island | MGK97290 | M | L | 0 | 1 | 1 | 0 | 0 | 0 | UMUT |
| 3 | Kinkazan Island | MGK99004 | M | L | 0 | 1 | 0 | 1 | 0 | 1 | UMUT |
| 3 | Kinkazan Island | MGK99006 | M | L | 0 | 1 | 0 | 1 | 0 | 1 | UMUT |
| 3 | Kinkazan Island | MGK99008 | F | L | 0 | 1 | 0 | 1 | 0 | 1 | UMUT |
| 3 | Kinkazan Island | MGK99010 | F | L | 0 | 1 | 0 | 1 | 0 | 1 | UMUT |
| 3 | Kinkazan Island | MGK99012 | M | L | 0 | 1 | 0 | 1 | 0 | 1 | UMUT |
| 3 | Kinkazan Island | MGK99013 | M | L | 0 | 1 | 1 | 0 | 0 | 1 | UMUT |
| 3 | Kinkazan Island | MGK99017 | F | L | 1 | 0 | 0 | 1 | 0 | 2 | UMUT |
| 3 | Kinkazan Island | MGK99023 | F | L | 0 | 1 | 0 | 1 | 0 | 1 | UMUT |
| 3 | Kinkazan Island | MG99026 | M | L | 0 | 1 | 1 | 0 | 0 | 1 | UMUT |
| 3 | Kinkazan Island | MG99028 | F | L | 0 | 1 | 0 | 1 | 0 | 1 | UMUT |
| 3 | Kinkazan Island | MGK99030 | M | L | 1 | 0 | 0 | 1 | 0 | 2 | UMUT |
| 3 | Kinkazan Island | MGK99033 | F | L | 0 | 1 | 0 | 1 | 0 | 1 | UMUT |
| 3 | Kinkazan Island | MGK99036 | F | L | 0 | 1 | 1 | 0 | 0 | 0 | UMUT |
| 3 | Kinkazan Island | MGK99037 | M | L | 0 | 1 | 1 | 0 | 0 | 0 | UMUT |
| 3 | Kinkazan Island | MGK99041 | M | L | 0 | 1 | 0 | 1 | 0 | 1 | UMUT |
| 3 | Kinkazan Island | MGK99042 | F | L | 0 | 1 | 0 | 1 | 0 | 1 | UMUT |
| 3 | Kinkazan Island | MGK99048 | M | L | 0 | 1 | 0 | 1 | 0 | 1 | UMUT |
| 3 | Kinkazan Island | MGK99052 | F | L | 0 | 1 | 0 | 1 | 0 | 1 | UMUT |
| 3 | Kinkazan Island | MGK99055 | F | L | 0 | 1 | 0 | 1 | 0 | 1 | UMUT |
| 3 | Kinkazan Island | MGK99063 | F | L | 0 | 1 | 0 | 1 | 0 | 1 | UMUT |
| 3 | Kinkazan Island | MGK99065 | F | L | 0 | 1 | 0 | 1 | 0 | 1 | UMUT |
| 3 | Kinkazan Island | MGK99071 | F | L | 0 | 1 | 0 | 1 | 0 | 1 | UMUT |
| 3 | Kinkazan Island | MGK99072 | F | L | 0 | 1 | 0 | 1 | 0 | 1 | UMUT |
| 3 | Kinkazan Island | MGK99073 | F | L | 0 | 1 | 0 | 1 | 0 | 1 | UMUT |
| 3 | Kinkazan Island | MGK00008 | F | L | 0 | 1 | 0 | 1 | 0 | 1 | UMUT |
| 3 | Kinkazan Island | MGK00014 | F | L | 0 | 1 | 1 | 0 | 0 | 0 | UMUT |
| 3 | Kinkazan Island | MGK00016 | M | L | 1 | 0 | 0 | 1 | 0 | 2 | UMUT |
| 3 | Kinkazan Island | MGK00021 | F | L | 0 | 1 | 1 | 0 | 0 | 0 | UMUT |
| 3 | Kinkazan Island | MGK00022 | F | L | 1 | 0 | 1 | 0 | 0 | 2.5 | UMUT |
| 3 | Kinkazan Island | MGK00026 | F | L | 0 | 1 | 0 | 1 | 0 | 1 | UMUT |
| 3 | Kinkazan Island | MGK00027 | F | L | 0 | 1 | 1 | 0 | 0 | 0 | UMUT |
| 3 | Kinkazan Island | MGK00029 | M | L | 0 | 1 | 0 | 1 | 0 | 1 | UMUT |
| 3 | Kinkazan Island | MGK00030 | F | L | 0 | 1 | 1 | 0 | 0 | 0 | UMUT |
| 3 | Kinkazan Island | MGK00041 | F | L | 0 | 1 | 0 | 1 | 0 | 1 | UMUT |
| 3 | Kinkazan Island | MG01012 | F | L | 0 | 1 | 1 | 0 | 0 | 0 | UMUT |
| 3 | Kinkazan Island | MG01022 | M | L | 0 | 1 | 1 | 0 | 0 | 0 | UMUT |
| 3 | Kinkazan Island | MG01023 | F | L | 0 | 1 | 1 | 0 | 0 | 0 | UMUT |
| 3 | Kinkazan Island | MGK01036 | F | L | 0 | 1 | 1 | 0 | 0 | 0 | UMUT |
| 3 | Kinkazan Island | MGK01040 | F | L | 0 | 1 | 1 | 0 | 0 | 0 | UMUT |
| 3 | Kinkazan Island | MGK01042 | M | L | 0 | 1 | 0 | 1 | 0 | 1 | UMUT |
| 3 | Kinkazan Island | MGK01047 | F | L | 0 | 1 | 0 | 1 | 0 | 1 | UMUT |
| 3 | Kinkazan Island | MGK01051 | F | L | 0 | 1 | 0 | 1 | 0 | 1 | UMUT |
| 3 | Kinkazan Island | MGK01065 | F | L | 0 | 1 | 0 | 1 | 0 | 1 | UMUT |
| 3 | Kinkazan Island | MGK01075 | F | L | 0 | 1 | 0 | 1 | 0 | 1 | UMUT |
| 3 | Kinkazan Island | MGK01077 | F | L | 0 | 1 | 1 | 0 | 0 | 0 | UMUT |
| 3 | Kinkazan Island | MG01084 | F | L | 0 | 1 | 0 | 1 | 0 | 1 | UMUT |
| 3 | Kinkazan Island | MGK01085 | F | L | 0 | 1 | 1 | 0 | 0 | 0 | UMUT |
| 3 | Kinkazan Island | MGK01086 | F | L | 0 | 1 | 1 | 0 | 0 | 0 | UMUT |
| 3 | Kinkazan Island | MG03023 | F | L | 0 | 1 | 0 | 1 | 0 | 1 | UMUT |
| 3 | Kinkazan Island | MG03051 | M | L | 0 | 1 | 0 | 1 | 0 | 1 | UMUT |
| 3 | Kinkazan Island | MG03052 | F | L | 0 | 1 | 0 | 1 | 0 | 1 | UMUT |
| 3 | Kinkazan Island | MG03059 | F | L | 0 | 1 | 0 | 1 | 0 | 1 | UMUT |
| 3 | Kinkazan Island | MG03066 | F | L | 0 | 1 | 0 | 1 | 0 | 1 | UMUT |
| 3 | Kinkazan Island | MG03070 | M | L | 0 | 1 | 1 | 0 | 0 | 0 | UMUT |
| 3 | Kinkazan Island | MG03101 | F | L | 0 | 1 | 0 | 1 | 0 | 1 | UMUT |
| 3 | Kinkazan Island | MG03108 | F | L | 0 | 1 | 0 | 1 | 0 | 1 | UMUT |
| 3 | Kinkazan Island | MG03123 | F | L | 0 | 1 | 0 | 1 | 0 | 1 | UMUT |
| 3 | Kinkazan Island | MG03125 | F | L | 0 | 1 | 0 | 1 | 0 | 1 | UMUT |
| 3 | Kinkazan Island | MG07006 | F | L | 0 | 1 | 1 | 0 | 0 | 0 | UMUT |
| 3 | Kinkazan Island | MG07009 | F | L | 0 | 1 | 0 | 1 | 0 | 1 | UMUT |
| 3 | Kinkazan Island | MG07013 | F | L | 0 | 1 | 0 | 1 | 0 | 1 | UMUT |
| 3 | Kinkazan Island | MG07028 | F | L | 0 | 1 | 1 | 0 | 0 | 0 | UMUT |
| 3 | Kinkazan Island | MG07029 | F | L | 1 | 0 | 0 | 1 | 0 | 2 | UMUT |
| 3 | Kinkazan Island | MG07030 | F | L | 0 | 1 | 0 | 1 | 0 | 1 | UMUT |
| 3 | Kinkazan Island | MG07032 | F | L | 0 | 1 | 0 | 1 | 0 | 1 | UMUT |
| 3 | Kinkazan Island | MG07033 | F | L | 0 | 1 | 1 | 0 | 0 | 0 | UMUT |
| 4 | Nikko | TG-0404 | F | L | 0 | 1 | 1 | 0 | 0 | 0 | UMUT |
| 4 | Nikko | TG-0405 | F | L | 0 | 1 | 0 | 1 | 0 | 1 | UMUT |
| 4 | Nikko | TG-0406 | F | L | 0 | 1 | 1 | 0 | 0 | 0 | UMUT |
| 4 | Nikko | TG-0408 | F | L | 0 | 1 | 0 | 1 | 0 | 1 | UMUT |
| 4 | Nikko | TG-0409 | F | L | 0 | 1 | 1 | 0 | 0 | 0 | UMUT |
| 4 | Nikko | TG-0410 | F | L | 0 | 1 | 0 | 1 | 0 | 1 | UMUT |
| 4 | Nikko | TG-0411 | F | L | 0 | 1 | 0 | 1 | 0 | 1 | UMUT |
| 4 | Nikko | TG-0412 | F | R | 0 | 1 | 0 | 1 | 0 | 1 | UMUT |
| 4 | Nikko | TG-0413 | F | L | 0 | 1 | 0 | 1 | 0 | 1 | UMUT |
| 4 | Nikko | TG-0414 | F | L | 0 | 1 | 1 | 0 | 0 | 0 | UMUT |
| 4 | Nikko | TG-0415 | F | L | 0 | 1 | 0 | 1 | 0 | 1 | UMUT |
| 4 | Nikko | TG-0416 | F | L | 0 | 1 | 1 | 0 | 0 | 0 | UMUT |
| 4 | Nikko | TG-0417 | F | L | 0 | 1 | 0 | 1 | 0 | 1 | UMUT |
| 4 | Nikko | TG-0418 | F | L | 0 | 1 | 0 | 1 | 0 | 1 | UMUT |
| 4 | Nikko | TG-0422 | F | L | 1 | 0 | 0 | 1 | 0 | 2 | UMUT |
| 4 | Nikko | M160 | M | L | 0 | 1 | 0 | 1 | 0 | 1 | TPM |
| 4 | Nikko | M161 | M | L | 0 | 1 | 1 | 0 | 0 | 0 | TPM |
| 4 | Nikko | M167 | F | L | 0 | 1 | 1 | 0 | 0 | 0 | TPM |
| 4 | Nikko | M171 | M | L | 1 | 0 | 1 | 0 | 0 | 2.5 | TPM |
| 4 | Nikko | M172 | M | L | 1 | 0 | 0 | 1 | 0 | 2 | TPM |
| 4 | Nikko | M180 | F | L | 0 | 1 | 0 | 1 | 0 | 1 | TPM |
| 4 | Nikko | M181 | F | L | 0 | 1 | 1 | 0 | 0 | 0 | TPM |
| 4 | Nikko | M191 | F | L | 0 | 1 | 1 | 0 | 0 | 0 | TPM |
| 4 | Nikko | M192 | M | R | 0 | 1 | 0 | 1 | 0 | 1 | TPM |
| 4 | Nikko | M193 | F | L | 0 | 1 | 1 | 0 | 0 | 0 | TPM |
| 4 | Nikko | M199 | M | L | 0 | 1 | 1 | 0 | 0 | 0 | TPM |
| 4 | Nikko | M324 | M | L | 0 | 1 | 0 | 1 | 0 | 1 | TPM |
| 4 | Nikko | M355 | M | L | 0 | 1 | 0 | 1 | 0 | 1 | TPM |
| 4 | Nikko | M363 | F | L | 0 | 1 | 1 | 0 | 0 | 0 | TPM |
| 4 | Nikko | M1683 | F | L | 0 | 1 | 0 | 1 | 0 | 1 | TPM |
| 4 | Nikko | M1895 | F | L | 0 | 1 | 0 | 1 | 0 | 1 | TPM |
| 4 | Nikko | M2357 | F | L | 0 | 1 | 0 | 1 | 0 | 1 | TPM |
| 4 | Nikko | M2359 | F | L | 0 | 1 | 1 | 0 | 0 | 0 | TPM |
| 4 | Nikko | M3648 | M | L | 0 | 1 | 0 | 1 | 0 | 1 | TPM |
| 4 | Nikko | M3650 | F | L | 0 | 1 | 0 | 1 | 0 | 1 | TPM |
| 4 | Nikko | M3773 | F | L | 0 | 1 | 1 | 0 | 0 | 0 | TPM |
| 4 | Nikko | M3788 | M | L | 0 | 1 | 0 | 1 | 0 | 1 | TPM |
| 4 | Nikko | M3863 | F | L | 0 | 1 | 1 | 0 | 0 | 0 | TPM |
| 4 | Nikko | M4145 | F |  | 0 | 1 | 1 | 0 | 0 | 0 | TPM |
| 4 | Nikko | M4146 | F |  | 0 | 1 | 1 | 0 | 0 | 0 | TPM |
| 4 | Nikko | M4161 | F |  | 0 | 1 | 1 | 0 | 0 | 0 | TPM |
| 4 | Nikko | M4164 | F |  | 0 | 1 | 1 | 0 | 0 | 0 | TPM |
| 4 | Nikko | M4167 | F |  | 0 | 1 | 1 | 0 | 0 | 0 | TPM |
| 5 | Ashio | TG-0401 | F | L | 0 | 1 | 0 | 1 | 0 | 1 | UMUT |
| 5 | Ashio | TG-0503 | F | L | 1 | 0 | 0 | 1 | 0 | 2 | UMUT |
| 5 | Ashio | TG-0511 | F | L | 0 | 1 | 1 | 0 | 0 | 0 | UMUT |
| 5 | Ashio | TG-0512 | F | L | 0 | 1 | 1 | 0 | 0 | 0 | UMUT |
| 5 | Ashio | TG-0513 | F | L | 0 | 1 | 0 | 1 | 0 | 1 | UMUT |
| 5 | Ashio | TG-0521 | F | R | 0 | 1 | 0 | 1 | 0 | 1 | UMUT |
| 5 | Ashio | M3796 | M | L | 1 | 0 | 0 | 1 | 0 | 2 | TPM |
| 6 | Okutama | TK-0508 | F | L | 1 | 0 | 0 | 1 | 0 | 2 | UMUT |
| 6 | Okutama | TK-0514 | F | L | 0 | 1 | 0 | 1 | 0 | 1 | UMUT |
| 6 | Okutama | TK-0515 | F | L | 0 | 1 | 0 | 1 | 0 | 1 | UMUT |
| 6 | Okutama | TK-0517 | F | L | 0 | 1 | 1 | 0 | 0 | 0 | UMUT |
| 6 | Okutama | TK-0519 | F | L | 0 | 1 | 1 | 0 | 0 | 0 | UMUT |
| 6 | Okutama | TK-0303 | F | L | 0 | 1 | 0 | 1 | 0 | 1 | UMUT |
| 6 | Okutama | TK-0503 | F | L | 0 | 1 | 0 | 1 | 0 | 1 | UMUT |
| 6 | Okutama | TK-0509 | F | L | 0 | 1 | 0 | 1 | 0 | 1 | UMUT |
| 6 | Okutama | TK-0510 | F | L | 0 | 1 | 1 | 0 | 0 | 0 | UMUT |
| 6 | Okutama | TK-0536 | F | L | 0 | 1 | 0 | 1 | 0 | 1 | UMUT |
| 6 | Okutama | TK-0507 | F | L | 0 | 1 | 1 | 0 | 0 | 0 | UMUT |
| 6 | Okutama | TK-0516 | F | L | 0 | 1 | 0 | 1 | 0 | 1 | UMUT |
| 6 | Okutama | TK-0523 | F | L | 0 | 1 | 1 | 0 | 0 | 0 | UMUT |
| 6 | Okutama | TK-0524 | F | L | 0 | 1 | 0 | 1 | 0 | 1 | UMUT |
| 6 | Okutama | TK-0530 | F | L | 0 | 1 | 1 | 0 | 0 | 0 | UMUT |
| 6 | Okutama | TK-0537 | F | L | 0 | 1 | 0 | 1 | 0 | 1 | UMUT |
| 6 | Okutama | TK-0302 | M | L | 0 | 1 | 0 | 1 | 0 | 1 | UMUT |
| 6 | Okutama | TK-0526 | M | L | 0 | 1 | 1 | 0 | 0 | 0 | UMUT |
| 7 | Boso Peninsula | 1075 | M | L | 0 | 1 | 0 | 1 | 0 | 1 | NHMIC |
| 7 | Boso Peninsula | 1076 | M | L | 0 | 1 | 0 | 1 | 0 | 1 | NHMIC |
| 7 | Boso Peninsula | 1077 | M | L | 0 | 1 | 0 | 1 | 0 | 1 | NHMIC |
| 7 | Boso Peninsula | 1150 | F | L | 0 | 1 | 0 | 1 | 0 | 1 | NHMIC |
| 7 | Boso Peninsula | 1156 | F | R | 0 | 1 | 0 | 1 | 0 | 1 | NHMIC |
| 7 | Boso Peninsula | 1158 | M | L | 0 | 1 | 1 | 0 | 0 | 0 | NHMIC |
| 7 | Boso Peninsula | 1163 | F | L | 0 | 1 | 1 | 0 | 0 | 0 | NHMIC |
| 7 | Boso Peninsula | 1165 | M | L | 0 | 1 | 0 | 1 | 0 | 1 | NHMIC |
| 7 | Boso Peninsula | 1260 | F | L | 0 | 1 | 0 | 1 | 0 | 1 | NHMIC |
| 7 | Boso Peninsula | 1262 | F | L | 0 | 1 | 0 | 1 | 0 | 1 | NHMIC |
| 7 | Boso Peninsula | 1268 | F | L | 0 | 1 | 0 | 1 | 0 | 1 | NHMIC |
| 7 | Boso Peninsula | 1276 | F | L | 0 | 1 | 1 | 0 | 0 | 0 | NHMIC |
| 7 | Boso Peninsula | 1278 | F | L | 0 | 1 | 1 | 0 | 0 | 0 | NHMIC |
| 7 | Boso Peninsula | 1291 | M | L | 0 | 1 | 1 | 0 | 0 | 0 | NHMIC |
| 7 | Boso Peninsula | 1292 | M | L | 0 | 1 | 1 | 0 | 0 | 0 | NHMIC |
| 7 | Boso Peninsula | 1297 | F | L | 0 | 1 | 1 | 0 | 0 | 0 | NHMIC |
| 7 | Boso Peninsula | 1298 | F | L | 0 | 1 | 1 | 0 | 0 | 0 | NHMIC |
| 7 | Boso Peninsula | 1307 | F | L | 0 | 1 | 1 | 0 | 0 | 0 | NHMIC |
| 7 | Boso Peninsula | 1308 | M | L | 0 | 1 | 1 | 0 | 0 | 0 | NHMIC |
| 7 | Boso Peninsula | 1314 | F | L | 0 | 1 | 1 | 0 | 0 | 0 | NHMIC |
| 7 | Boso Peninsula | 1317 | F | L | 0 | 1 | 0 | 1 | 0 | 1 | NHMIC |
| 7 | Boso Peninsula | 1318 | F | L | 0 | 1 | 0 | 1 | 0 | 1 | NHMIC |
| 7 | Boso Peninsula | 1319 | F | L | 0 | 1 | 0 | 1 | 0 | 1 | NHMIC |
| 7 | Boso Peninsula | 1322 | F | L | 0 | 1 | 1 | 0 | 0 | 0 | NHMIC |
| 7 | Boso Peninsula | 1330 | F | L | 0 | 1 | 1 | 0 | 0 | 0 | NHMIC |
| 7 | Boso Peninsula | 1332 | F | L | 0 | 1 | 1 | 0 | 0 | 0 | NHMIC |
| 7 | Boso Peninsula | 1345 | F | L | 0 | 1 | 0 | 1 | 0 | 1 | NHMIC |
| 7 | Boso Peninsula | 1350 | F | L | 0 | 1 | 1 | 0 | 0 | 0 | NHMIC |
| 7 | Boso Peninsula | 1356 | F | L | 0 | 1 | 0 | 1 | 0 | 1 | NHMIC |
| 7 | Boso Peninsula | 1359 | F | L | 0 | 1 | 1 | 0 | 0 | 0 | NHMIC |
| 7 | Boso Peninsula | 1371 | F | L | 0 | 1 | 0 | 1 | 0 | 1 | NHMIC |
| 7 | Boso Peninsula | 1375 | F | L | 0 | 1 | 0 | 1 | 0 | 1 | NHMIC |
| 7 | Boso Peninsula | 1388 | F | L | 0 | 1 | 0 | 1 | 0 | 1 | NHMIC |
| 7 | Boso Peninsula | 1391 | F | L | 0 | 1 | 0 | 1 | 0 | 1 | NHMIC |
| 7 | Boso Peninsula | 1400 | F | R | 0 | 1 | 0 | 1 | 0 | 1 | NHMIC |
| 7 | Boso Peninsula | 1428 | F | L | 0 | 1 | 0 | 1 | 0 | 1 | NHMIC |
| 7 | Boso Peninsula | 1430 | F | L | 0 | 1 | 0 | 1 | 0 | 1 | NHMIC |
| 7 | Boso Peninsula | 1433 | F | L | 0 | 1 | 0 | 1 | 0 | 1 | NHMIC |
| 7 | Boso Peninsula | 1456 | F | L | 0 | 1 | 0 | 1 | 0 | 1 | NHMIC |
| 7 | Boso Peninsula | 1458 | F | L | 0 | 1 | 1 | 0 | 0 | 0 | NHMIC |
| 7 | Boso Peninsula | 1461 | F | L | 0 | 1 | 0 | 1 | 0 | 1 | NHMIC |
| 7 | Boso Peninsula | 1465 | F | L | 0 | 1 | 1 | 0 | 0 | 0 | NHMIC |
| 7 | Boso Peninsula | 1492 | M | L | 0 | 1 | 1 | 0 | 0 | 0 | NHMIC |
| 7 | Boso Peninsula | 1493 | F | L | 0 | 1 | 1 | 0 | 0 | 0 | NHMIC |
| 7 | Boso Peninsula | 1503 | F | L | 0 | 1 | 1 | 0 | 0 | 0 | NHMIC |
| 7 | Boso Peninsula | 1505 | M | L | 0 | 1 | 0 | 1 | 0 | 1 | NHMIC |
| 7 | Boso Peninsula | 1506 | M | L | 0 | 1 | 1 | 0 | 0 | 0 | NHMIC |
| 7 | Boso Peninsula | 1509 | M | L | 0 | 1 | 1 | 0 | 0 | 0 | NHMIC |
| 7 | Boso Peninsula | 1510 | F | L | 0 | 1 | 1 | 0 | 0 | 0 | NHMIC |
| 7 | Boso Peninsula | 1579 | M | R | 0 | 1 | 0 | 1 | 0 | 1 | NHMIC |
| 7 | Boso Peninsula | 1586 | F | L | - | - | 0 | 1 | 0 | 1 | NHMIC |
| 7 | Boso Peninsula | 1587 | M | L | 0 | 1 | 1 | 0 | 0 | 0 | NHMIC |
| 7 | Boso Peninsula | 1588 | M | L | 0 | 1 | 1 | 0 | 0 | 0 | NHMIC |
| 7 | Boso Peninsula | 1593 | M | L | 0 | 1 | 1 | 0 | 0 | 0 | NHMIC |
| 7 | Boso Peninsula | 1594 | F | L | 0 | 1 | 1 | 0 | 0 | 0 | NHMIC |
| 7 | Boso Peninsula | 1600 | F | L | 0 | 1 | 1 | 0 | 0 | 0 | NHMIC |
| 7 | Boso Peninsula | 1606 | M | L | 0 | 1 | 1 | 0 | 0 | 0 | NHMIC |
| 7 | Boso Peninsula | 1614 | M | L | 0 | 1 | 1 | 0 | 0 | 0 | NHMIC |
| 7 | Boso Peninsula | 1681 | M | L | 0 | 1 | 1 | 0 | 0 | 0 | NHMIC |
| 7 | Boso Peninsula | 1733 | M | L | 0 | 1 | 1 | 0 | 0 | 0 | NHMIC |
| 7 | Boso Peninsula | 1734 | M | L | 0 | 1 | 1 | 0 | 0 | 0 | NHMIC |
| 7 | Boso Peninsula | 1742 | M | L | 0 | 1 | 0 | 1 | 0 | 1 | NHMIC |
| 7 | Boso Peninsula | 1744 | F | L | 0 | 1 | 0 | 1 | 0 | 1 | NHMIC |
| 7 | Boso Peninsula | 1778 | M | L | 0 | 1 | 0 | 1 | 0 | 1 | NHMIC |
| 7 | Boso Peninsula | 1789 | M | L | 0 | 1 | 1 | 0 | 0 | 0 | NHMIC |
| 7 | Boso Peninsula | 1793 | F | L | 0 | 1 | 1 | 0 | 0 | 0 | NHMIC |
| 7 | Boso Peninsula | 1795 | F | L | 0 | 1 | 1 | 0 | 0 | 0 | NHMIC |
| 7 | Boso Peninsula | 1800 | F | L | 0 | 1 | 1 | 0 | 0 | 0 | NHMIC |
| 7 | Boso Peninsula | 1862 | M | L | 0 | 1 | 1 | 0 | 0 | 0 | NHMIC |
| 7 | Boso Peninsula | 1908 | M | R | 0 | 1 | 0 | 1 | 0 | 1 | NHMIC |
| 7 | Boso Peninsula | 1920 | M | L | 0 | 1 | 0 | 1 | 0 | 1 | NHMIC |
| 7 | Boso Peninsula | 1924 | M | L | 0 | 1 | 0 | 1 | 0 | 1 | NHMIC |
| 7 | Boso Peninsula | 1935 | M | L | 0 | 1 | 0 | 1 | 0 | 1 | NHMIC |
| 7 | Boso Peninsula | 1962 | M | L | 0 | 1 | 0 | 1 | 0 | 1 | NHMIC |
| 7 | Boso Peninsula | 1971 | M | L | 0 | 1 | 0 | 1 | 0 | 1 | NHMIC |
| 7 | Boso Peninsula | 2000 | M | L | 0 | 1 | 0 | 1 | 0 | 1 | NHMIC |
| 7 | Boso Peninsula | 2040 | M | L | 0 | 1 | 0 | 1 | 0 | 1 | NHMIC |
| 7 | Boso Peninsula | 2041 | M | L | 0 | 1 | 1 | 0 | 0 | 0 | NHMIC |
| 7 | Boso Peninsula | 2049 | M | L | 0 | 1 | 1 | 0 | 0 | 0 | NHMIC |
| 7 | Boso Peninsula | 2050 | M | L | 0 | 1 | 1 | 0 | 0 | 0 | NHMIC |
| 7 | Boso Peninsula | 2051 | M | L | 0 | 1 | 1 | 0 | 0 | 0 | NHMIC |
| 8 | Izu Peninsula | SO-0402 | F | L | 0 | 1 | 1 | 0 | 0 | 0 | UMUT |
| 8 | Izu Peninsula | SO-0407 | F | L | 0 | 1 | 1 | 0 | 0 | 0 | UMUT |
| 8 | Izu Peninsula | SO-0414 | F | L | 0 | 1 | 1 | 0 | 0 | 0 | UMUT |
| 8 | Izu Peninsula | SO-0418 | F | L | 0 | 1 | 1 | 0 | 0 | 0 | UMUT |
| 8 | Izu Peninsula | SO-0419 | F | L | 0 | 1 | 0 | 1 | 0 | 1 | UMUT |
| 8 | Izu Peninsula | SO-0422 | F | L | 0 | 1 | 0 | 1 | 0 | 1 | UMUT |
| 8 | Izu Peninsula | SO-0426 | F | L | 0 | 1 | 1 | 0 | 0 | 0 | UMUT |
| 8 | Izu Peninsula | SO-0427 | F | L | 0 | 1 | 0 | 1 | 0 | 1 | UMUT |
| 8 | Izu Peninsula | SO-0428 | F | L | 0 | 1 | 1 | 0 | 0 | 0 | UMUT |
| 8 | Izu Peninsula | SO-0434 | F | L | 0 | 1 | 0 | 1 | 0 | 1 | UMUT |
| 8 | Izu Peninsula | SO-0436 | F | L | 0 | 1 | 0 | 1 | 0 | 1 | UMUT |
| 8 | Izu Peninsula | SO-0441 | F | L | 0 | 1 | 0 | 1 | 0 | 1 | UMUT |
| 8 | Izu Peninsula | SO-0442 | F | L | 0 | 1 | 0 | 1 | 0 | 1 | UMUT |
| 8 | Izu Peninsula | SO-0445 | F | L | 0 | 1 | 1 | 0 | 0 | 0 | UMUT |
| 8 | Izu Peninsula | SO-0446 | F | L | 0 | 1 | 0 | 1 | 0 | 1 | UMUT |
| 8 | Izu Peninsula | SO-0448 | F | L | 0 | 1 | 1 | 0 | 0 | 0 | UMUT |
| 8 | Izu Peninsula | SO-0461 | F | L | 0 | 1 | 0 | 1 | 0 | 1 | UMUT |
| 8 | Izu Peninsula | SO-0463 | F | L | 0 | 1 | 1 | 0 | 0 | 0 | UMUT |
| 8 | Izu Peninsula | SO-0470 | F | R | 0 | 1 | 0 | 1 | 0 | 1 | UMUT |
| 8 | Izu Peninsula | SO-0471 | F | L | 0 | 1 | 1 | 0 | 0 | 0 | UMUT |
| 8 | Izu Peninsula | SO-0305 | F | L | 0 | 1 | 1 | 0 | 0 | 0 | UMUT |
| 8 | Izu Peninsula | SO-0311 | F | L | 0 | 1 | 0 | 1 | 0 | 1 | UMUT |
| 8 | Izu Peninsula | SO-0307 | M | L | 0 | 1 | 0 | 1 | 0 | 1 | UMUT |
| 8 | Izu Peninsula | SO-0317 | M | L | 0 | 1 | 0 | 1 | 0 | 1 | UMUT |
| 8 | Izu Peninsula | SO-0412 | M | L | 0 | 1 | 1 | 0 | 0 | 0 | UMUT |
| 8 | Izu Peninsula | SO-04115 | M | L | 0 | 1 | 0 | 1 | 0 | 1 | UMUT |
| 9 | Yamanashi | YN-0002 | F | L | 0 | 1 | 1 | 0 | 0 | 0 | UMUT |
| 9 | Yamanashi | YN-0102 | F | L | 0 | 1 | 1 | 0 | 0 | 0 | UMUT |
| 9 | Yamanashi | YN-0103 | F | L | 0 | 1 | 1 | 0 | 0 | 0 | UMUT |
| 9 | Yamanashi | YN-0501 | F | L | 0 | 1 | 0 | 1 | 0 | 1 | UMUT |
| 9 | Yamanashi | YN-0505 | M | L | 0 | 1 | 0 | 1 | 0 | 1 | UMUT |
| 9 | Yamanashi | YN-0506 | M | L | 0 | 1 | 0 | 1 | 0 | 1 | UMUT |
| 9 | Yamanashi | YN-0507 | M | L | 0 | 1 | 0 | 1 | 0 | 1 | UMUT |
| 9 | Yamanashi | YN-0508 | F | L | 0 | 1 | 0 | 1 | 0 | 1 | UMUT |
| 9 | Yamanashi | YN-0509 | F | L | 0 | 1 | 0 | 1 | 0 | 1 | UMUT |
| 9 | Yamanashi | YN-0510 | M | L | 0 | 1 | 0 | 1 | 0 | 1 | UMUT |
| 9 | Yamanashi | YN-0512 | F | L | 0 | 1 | 1 | 0 | 0 | 0 | UMUT |
| 9 | Yamanashi | YN-0514 | M | L | 0 | 1 | 0 | 1 | 0 | 1 | UMUT |
| 9 | Yamanashi | YN-0519 | F | L | 0 | 1 | 1 | 0 | 0 | 0 | UMUT |
| 9 | Yamanashi | YN-0522 | M | L | 0 | 1 | 1 | 0 | 0 | 0 | UMUT |
| 9 | Yamanashi | YN-0523 | F | L | 1 | 0 | 0 | 1 | 0 | 2 | UMUT |
| 10 | Nara Park | P608 | F | L | 0 | 1 | 0 | 1 | 0 | 1 | HUM |
| 10 | Nara Park | 434 | F | R | 0 | 1 | 0 | 1 | 0 | 1 | HUM |
| 10 | Nara Park | 572 | F | L | 0 | 1 | 1 | 0 | 0 | 0 | HUM |
| 10 | Nara Park | 570 | F | L | 0 | 1 | 1 | 0 | 0 | 0 | HUM |
| 10 | Nara Park | 607 | F | R | 0 | 1 | 1 | 0 | 0 | 0 | HUM |
| 10 | Nara Park | 601 | F | L | 0 | 1 | 1 | 0 | 0 | 0 | HUM |
| 10 | Nara Park | 442 | F | L | 0 | 1 | 1 | 0 | 0 | 0 | HUM |
| 10 | Nara Park | 576 | M | L | 0 | 1 | 1 | 0 | 0 | 0 | HUM |
| 10 | Nara Park | 582 | M | L | 0 | 1 | 0 | 1 | 0 | 1 | HUM |
| 10 | Nara Park | 422 | M | L | 0 | 1 | 1 | 0 | 0 | 0 | HUM |
| 10 | Nara Park | 457 | M | L | 0 | 1 | 1 | 0 | 0 | 0 | HUM |
| 10 | Nara Park | 453 | M | L | 0 | 1 | 1 | 0 | 0 | 0 | HUM |
| 10 | Nara Park | 615 | M | L | 0 | 1 | 1 | 0 | 0 | 0 | HUM |
| 10 | Nara Park | 621 | M | L | 0 | 1 | 1 | 0 | 0 | 0 | HUM |
| 10 | Nara Park | 452 | M | L | 0 | 1 | 0 | 1 | 0 | 1 | HUM |
| 10 | Nara Park | 770 | F | L | 1 | 0 | 0 | 1 | 0 | 2 | HUM |
| 10 | Nara Park | UnknownF | F | L | 1 | 0 | 0 | 1 | 0 | 2 | HUM |
| 10 | Nara Park | UnknownM | M | L | 0 | 1 | 1 | 0 | 0 | 0 | HUM |
| 10 | Nara Park | 420 | M | L | 0 | 1 | 0 | 1 | 0 | 1 | HUM |
| 10 | Nara Park | 611 | M | L | 0 | 1 | 1 | 0 | 0 | 0 | HUM |
| 10 | Nara Park | 424 | M | L | 0 | 1 | 0 | 1 | 0 | 1 | HUM |
| 10 | Nara Park | 619 | M | L | 0 | 1 | 1 | 0 | 0 | 0 | HUM |
| 10 | Nara Park | 792 | M | L | 0 | 1 | 0 | 1 | 0 | 1 | HUM |
| 10 | Nara Park | 451 | M | L | 0 | 1 | 1 | 0 | 0 | 0 | HUM |
| 10 | Nara Park | 822 | M | L | 0 | 1 | 0 | 1 | 0 | 1 | HUM |
| 10 | Nara Park | 585 | M | L | 1 | 0 | 0 | 1 | 0 | 2 | HUM |
| 10 | Nara Park | 449 | M | L | 0 | 1 | 1 | 0 | 0 | 0 | HUM |
| 10 | Nara Park | 441 | F | L | 0 | 1 | 0 | 1 | 0 | 1 | HUM |
| 10 | Nara Park | 781 | F | L | 0 | 1 | 1 | 0 | 0 | 0 | HUM |
| 10 | Nara Park | 405 | F | L | 0 | 1 | 1 | 0 | 0 | 0 | HUM |
| 10 | Nara Park | 798 | M | L | 0 | 1 | 1 | 0 | 0 | 0 | HUM |
| 11 | Shimane | SN-0302 | M | L | 0 | 1 | 0 | 1 | 0 | 1 | UMUT |
| 11 | Shimane | SN-0305 | F | L | 0 | 1 | 0 | 1 | 0 | 1 | UMUT |
| 11 | Shimane | SN-0310 | F | L | 0 | 1 | 1 | 0 | 0 | 0 | UMUT |
| 11 | Shimane | SN-0313 | M | L | 0 | 1 | 1 | 0 | 0 | 0 | UMUT |
| 11 | Shimane | SN-0319 | F | L | 0 | 1 | 1 | 0 | 0 | 0 | UMUT |
| 11 | Shimane | SN-0401 | M | L | 0 | 1 | 1 | 0 | 0 | 0 | UMUT |
| 11 | Shimane | SN-0402 | F | L | 0 | 1 | 1 | 0 | 0 | 0 | UMUT |
| 11 | Shimane | SN-0403 | F | L | 0 | 1 | 1 | 0 | 0 | 0 | UMUT |
| 11 | Shimane | SN-0405 | M | L | 0 | 1 | 1 | 0 | 0 | 0 | UMUT |
| 11 | Shimane | SN-0407 | M | L | 1 | 0 | 0 | 1 | 0 | 2 | UMUT |
| 11 | Shimane | SN-0408 | F | L | 0 | 1 | 1 | 0 | 0 | 0 | UMUT |
| 11 | Shimane | SN-0410 | F | L | 0 | 1 | 1 | 0 | 0 | 0 | UMUT |
| 11 | Shimane | SN-0411 | M | L | 0 | 1 | 1 | 0 | 0 | 0 | UMUT |
| 11 | Shimane | SN-0412 | F | L | 0 | 1 | 0 | 1 | 0 | 1 | UMUT |
| 11 | Shimane | SN-0413 | F | L | 0 | 1 | 0 | 1 | 0 | 1 | UMUT |
| 11 | Shimane | SN-0414 | M | L | 0 | 1 | 1 | 0 | 0 | 0 | UMUT |
| 11 | Shimane | SN-0415 | F | L | 0 | 1 | 1 | 0 | 0 | 0 | UMUT |
| 11 | Shimane | SN-0416 | M | L | 0 | 1 | 1 | 0 | 0 | 0 | UMUT |
| 11 | Shimane | SN-0417 | M | L | 0 | 1 | 1 | 0 | 0 | 0 | UMUT |
| 11 | Shimane | SN-0418 | M | L | 0 | 1 | 0 | 1 | 0 | 1 | UMUT |
| 11 | Shimane | SN-0419 | M | L | 0 | 1 | 0 | 1 | 0 | 1 | UMUT |
| 11 | Shimane | SN-0420 | M | L | 0 | 1 | 1 | 0 | 0 | 0 | UMUT |
| 11 | Shimane | SN-0421 | F | L | 0 | 1 | 0 | 1 | 0 | 1 | UMUT |
| 11 | Shimane | SN-0422 | F | L | 0 | 1 | 1 | 0 | 0 | 0 | UMUT |
| 11 | Shimane | SN-0423 | M | L | 0 | 1 | 0 | 1 | 0 | 1 | UMUT |
| 11 | Shimane | SN0424 | M | L | 1 | 0 | 0 | 1 | 0 | 2 | UMUT |
| 11 | Shimane | SN-0426 | F | L | 0 | 1 | 1 | 0 | 0 | 0 | UMUT |
| 11 | Shimane | SN-0427 | F | L | 0 | 1 | 1 | 0 | 0 | 0 | UMUT |
| 11 | Shimane | SN-0432 | M | L | 0 | 1 | 1 | 0 | 0 | 0 | UMUT |
| 11 | Shimane | SN-0434 | F | L | 0 | 1 | 0 | 1 | 0 | 1 | UMUT |
| 12 | Yamaguchi | YG-9903 | F | L | 0 | 1 | 0 | 1 | 0 | 1 | UMUT |
| 12 | Yamaguchi | YG-9904 | F | L | 0 | 1 | 1 | 0 | 0 | 0 | UMUT |
| 12 | Yamaguchi | YG-9905 | M | L | 0 | 1 | 0 | 1 | 0 | 1 | UMUT |
| 12 | Yamaguchi | YG-9906 | M | L | 0 | 1 | 0 | 1 | 0 | 1 | UMUT |
| 12 | Yamaguchi | YG-9907 | M | L | 0 | 1 | 1 | 0 | 0 | 0 | UMUT |
| 12 | Yamaguchi | YG-9913 | F | L | 0 | 1 | 0 | 1 | 0 | 1 | UMUT |
| 12 | Yamaguchi | YG-9914 | F | L | 0 | 1 | 1 | 0 | 0 | 0 | UMUT |
| 12 | Yamaguchi | YG-9917 | M | L | 0 | 1 | 1 | 0 | 0 | 0 | UMUT |
| 12 | Yamaguchi | YG-9918 | M | L | 0 | 1 | 1 | 0 | 0 | 0 | UMUT |
| 12 | Yamaguchi | YG-9922 | F | L | 0 | 1 | 1 | 0 | 0 | 0 | UMUT |
| 12 | Yamaguchi | YG-9924 | M | L | 0 | 1 | 0 | 1 | 0 | 1 | UMUT |
| 12 | Yamaguchi | YG-9925 | M | L | 0 | 1 | 0 | 1 | 0 | 1 | UMUT |
| 12 | Yamaguchi | YG-0401 | F | L | 0 | 1 | 0 | 1 | 0 | 1 | UMUT |
| 12 | Yamaguchi | YG-0402 | F | L | 0 | 1 | 1 | 0 | 0 | 0 | UMUT |
| 12 | Yamaguchi | YG-0403 | F | L | 0 | 1 | 1 | 0 | 0 | 0 | UMUT |
| 12 | Yamaguchi | YG-0404 | M | L | 0 | 1 | 1 | 0 | 0 | 0 | UMUT |
| 12 | Yamaguchi | YG-0405 | F | L | 0 | 1 | 1 | 0 | 0 | 0 | UMUT |
| 12 | Yamaguchi | YG-0409 | F | L | 0 | 1 | 1 | 0 | 0 | 0 | UMUT |
| 12 | Yamaguchi | YG-0410 | F | L | 0 | 1 | 0 | 1 | 0 | 1 | UMUT |
| 12 | Yamaguchi | YG-0411 | F | L | 0 | 1 | 0 | 1 | 0 | 1 | UMUT |
| 12 | Yamaguchi | YG-0412 | F | L | 0 | 1 | 0 | 1 | 0 | 1 | UMUT |
| 12 | Yamaguchi | YG-0416 | M | L | 0 | 1 | 1 | 0 | 0 | 0 | UMUT |
| 12 | Yamaguchi | YG-0417 | M | L | 0 | 1 | 0 | 1 | 0 | 1 | UMUT |
| 12 | Yamaguchi | YG-0420 | M | L | 0 | 1 | 0 | 1 | 0 | 1 | UMUT |
| 12 | Yamaguchi | YG-0421 | F | L | 0 | 1 | 1 | 0 | 0 | 0 | UMUT |
| 12 | Yamaguchi | YG-0422 | M | L | 0 | 1 | 0 | 1 | 0 | 1 | UMUT |
| 12 | Yamaguchi | YG-0424 | M | L | 0 | 1 | 1 | 0 | 0 | 0 | UMUT |
| 12 | Yamaguchi | YG-0425 | M | L | 0 | 1 | 0 | 1 | 0 | 1 | UMUT |
| 12 | Yamaguchi | YG-0427 | F | L | 0 | 1 | 0 | 1 | 0 | 1 | UMUT |
| 12 | Yamaguchi | YG-0428 | M | L | 1 | 0 | 1 | 0 | 0 | 2.5 | UMUT |
| 12 | Yamaguchi | YG-0430 | F | L | 0 | 1 | 1 | 0 | 0 | 0 | UMUT |
| 12 | Yamaguchi | YG-0431 | F | L | 0 | 1 | 0 | 1 | 0 | 1 | UMUT |
| 12 | Yamaguchi | YG-0432 | M | L | 0 | 1 | 1 | 0 | 0 | 0 | UMUT |
| 13 | Fukuoka | FO-0301 | M | L | 0 | 1 | 1 | 0 | 0 | 0 | UMUT |
| 13 | Fukuoka | FO-0401 | F | L | 0 | 1 | 1 | 0 | 0 | 0 | UMUT |
| 13 | Fukuoka | FO-0403 | F | L | 0 | 1 | 0 | 1 | 0 | 1 | UMUT |
| 13 | Fukuoka | FO-0404 | F | L | 0 | 1 | 0 | 1 | 0 | 1 | UMUT |
| 13 | Fukuoka | FO-0405 | F | L | 0 | 1 | 1 | 0 | 0 | 0 | UMUT |
| 13 | Fukuoka | FO-0406 | M | L | 0 | 1 | 1 | 0 | 0 | 0 | UMUT |
| 13 | Fukuoka | FO-0408 | F | L | 0 | 1 | 1 | 0 | 0 | 0 | UMUT |
| 13 | Fukuoka | FO-0409 | F | L | 0 | 1 | 1 | 0 | 0 | 0 | UMUT |
| 13 | Fukuoka | FO-0410 | M | L | 0 | 1 | 1 | 0 | 0 | 0 | UMUT |
| 13 | Fukuoka | FO-0411 | F | L | 0 | 1 | 0 | 1 | 0 | 1 | UMUT |
| 13 | Fukuoka | FO-0412 | M | L | 0 | 1 | 1 | 0 | 0 | 0 | UMUT |
| 13 | Fukuoka | FO-0413 | M | L | 0 | 1 | 0 | 1 | 0 | 1 | UMUT |
| 13 | Fukuoka | FO-0414 | F | L | 0 | 1 | 1 | 0 | 0 | 0 | UMUT |
| 13 | Fukuoka | FO-0415 | M | L | 0 | 1 | 0 | 1 | 0 | 1 | UMUT |
| 13 | Fukuoka | FO-0427 | F | L | 0 | 1 | 0 | 1 | 0 | 1 | UMUT |
| 13 | Fukuoka | FO-0428 | M | L | 0 | 1 | 0 | 1 | 0 | 1 | UMUT |
| 13 | Fukuoka | FO-0429 | F | L | 0 | 1 | 1 | 0 | 0 | 0 | UMUT |
| 13 | Fukuoka | FO-0430 | F | L | 0 | 1 | 0 | 1 | 0 | 1 | UMUT |
| 13 | Fukuoka | FO-0437 | F | L | 0 | 1 | 1 | 0 | 0 | 0 | UMUT |
| 13 | Fukuoka | FO-0438 | F | L | 0 | 1 | 0 | 1 | 0 | 1 | UMUT |
| 15 | Tsushima | T81♂9 | M | L | 0 | 1 | 1 | 0 | 0 | 0 | HUM |
| 15 | Tsushima | T81♂10 | M | L | 0 | 1 | 1 | 0 | 0 | 0 | HUM |
| 15 | Tsushima | T81♂11 | M | L | 0 | 1 | 1 | 0 | 0 | 0 | HUM |
| 15 | Tsushima | T81♂12 | M | L | 0 | 1 | 1 | 0 | 0 | 0 | HUM |
| 15 | Tsushima | T81♂13 | M | L | 0 | 1 | 0 | 1 | 0 | 1 | HUM |
| 15 | Tsushima | T81♂14 | M | L | 0 | 1 | 1 | 0 | 0 | 0 | HUM |
| 15 | Tsushima | T81♂15 | M | L | 0 | 1 | 1 | 0 | 0 | 0 | HUM |
| 15 | Tsushima | T81♂16 | M | L | 0 | 1 | 0 | 1 | 0 | 1 | HUM |
| 15 | Tsushima | T81♂17 | M | L | 0 | 1 | 1 | 0 | 0 | 0 | HUM |
| 15 | Tsushima | T81♀5 | F | L | 0 | 1 | 1 | 0 | 0 | 0 | HUM |
| 15 | Tsushima | T81♀6 | F | L | 0 | 1 | 1 | 0 | 0 | 0 | HUM |
| 15 | Tsushima | T81♀7 | F | L | 0 | 1 | 1 | 0 | 0 | 0 | HUM |
| 15 | Tsushima | T81♀8 | F | L | 0 | 1 | 1 | 0 | 0 | 0 | HUM |
| 15 | Tsushima | 12 | F | L | 0 | 1 | 0 | 1 | 0 | 1 | HUM |
| 15 | Tsushima | 13 | F | L | 0 | 1 | 1 | 0 | 0 | 0 | HUM |
| 15 | Tsushima | 14 | F | L | 0 | 1 | 1 | 0 | 0 | 0 | HUM |
| 15 | Tsushima | 21 | F | L | 0 | 1 | 1 | 0 | 0 | 0 | HUM |
| 15 | Tsushima | 22 | F | L | 0 | 1 | 1 | 0 | 0 | 0 | HUM |
| 15 | Tsushima | 27 | F | L | 0 | 1 | 1 | 0 | 0 | 0 | HUM |
| 15 | Tsushima | 43 | F | L | 0 | 1 | 1 | 0 | 0 | 0 | HUM |
| 15 | Tsushima | 53 | F | L | 0 | 1 | 1 | 0 | 0 | 0 | HUM |
| 15 | Tsushima | 55 | F | L | 0 | 1 | 1 | 0 | 0 | 0 | HUM |
| 15 | Tsushima | 57 | F | L | 0 | 1 | 1 | 0 | 0 | 0 | HUM |
| 15 | Tsushima | 60 | F | L | 0 | 1 | 1 | 0 | 0 | 0 | HUM |
| 15 | Tsushima | 76 | F | L | 0 | 1 | 1 | 0 | 0 | 0 | HUM |
| 15 | Tsushima | 78 | F | L | 0 | 1 | 1 | 0 | 0 | 0 | HUM |
| 15 | Tsushima | 79 | F | L | 0 | 1 | 1 | 0 | 0 | 0 | HUM |
| 15 | Tsushima | 84 | F | L | 0 | 1 | 1 | 0 | 0 | 0 | HUM |
| 15 | Tsushima | 87 | M | L | 0 | 1 | 0 | 1 | 0 | 1 | HUM |
| 15 | Tsushima | 88 | M | R | 0 | 1 | 1 | 0 | 0 | 0 | HUM |
| 15 | Tsushima | 89 | F | L | 0 | 1 | 1 | 0 | 0 | 0 | HUM |
| 15 | Tsushima | 2 | F | L | 0 | 1 | 1 | 0 | 0 | 0 | HUM |
| 15 | Tsushima | 92 | M | L | 0 | 1 | 1 | 0 | 0 | 0 | HUM |
| 15 | Tsushima | 94 | F | L | 0 | 1 | 1 | 0 | 0 | 0 | HUM |
| 15 | Tsushima | 95 | F | L | 0 | 1 | 1 | 0 | 0 | 0 | HUM |
| 15 | Tsushima | 96 | M | L | 0 | 1 | 1 | 0 | 0 | 0 | HUM |
| 15 | Tsushima | 100 | F | L | 0 | 1 | 1 | 0 | 0 | 0 | HUM |
| 15 | Tsushima | 103 | F | L | 0 | 1 | 1 | 0 | 0 | 0 | HUM |
| 15 | Tsushima | 104 | F | L | 0 | 1 | 1 | 0 | 0 | 0 | HUM |
| 15 | Tsushima | 106 | M | L | 0 | 1 | 1 | 0 | 0 | 0 | HUM |
| 15 | Tsushima | 110 | M | L | 0 | 1 | 1 | 0 | 0 | 0 | HUM |
| 15 | Tsushima | 111 | M | R | 0 | 1 | 1 | 0 | 0 | 0 | HUM |
| 15 | Tsushima | Unknown1 | F | L | 0 | 1 | 1 | 0 | 0 | 0 | HUM |
| 15 | Tsushima | Unknown2 | F | L | 0 | 1 | 1 | 0 | 0 | 0 | HUM |
| 15 | Tsushima | Unknown3 | F | L | 0 | 1 | 1 | 0 | 0 | 0 | HUM |
| 15 | Tsushima | M381 | M | L | 0 | 1 | 1 | 0 | 0 | 0 | TPM |
| 15 | Tsushima | M384 | F | L | 0 | 1 | 1 | 0 | 0 | 0 | TPM |
| 15 | Tsushima | M385 | M | L | 0 | 1 | 1 | 0 | 0 | 0 | TPM |
| 15 | Tsushima | M387 | M | L | 0 | 1 | 1 | 0 | 0 | 0 | TPM |
| 15 | Tsushima | M389 | M | L | 0 | 1 | 1 | 0 | 0 | 0 | TPM |
| 15 | Tsushima | M391 | F | L | 0 | 1 | 1 | 0 | 0 | 0 | TPM |
| 15 | Tsushima | M393 | F | L | 0 | 1 | 1 | 0 | 0 | 0 | TPM |
| 15 | Tsushima | M397 | M | L | 0 | 1 | 1 | 0 | 0 | 0 | TPM |
| 15 | Tsushima | M398 | M | L | 0 | 1 | 1 | 0 | 0 | 0 | TPM |
| 15 | Tsushima | M399 | M | L | 0 | 1 | 1 | 0 | 0 | 0 | TPM |
| 15 | Tsushima | M1119 | M | L | 0 | 1 | 1 | 0 | 0 | 0 | TPM |
| 16 | Yakushima Island | KGSY-0401 | F | L | 0 | 1 | 0 | 1 | 0 | 1 | UMUT |
| 16 | Yakushima Island | KGSY-0504 | M | L | 0 | 1 | 1 | 0 | 0 | 0 | UMUT |
| 16 | Yakushima Island | KGSY-0508 | F | L | 0 | 1 | 1 | 0 | 0 | 0 | UMUT |
| 16 | Yakushima Island | KGSY-0509 | F | L | 0 | 1 | 1 | 0 | 0 | 0 | UMUT |
| 16 | Yakushima Island | KGSY-0511 | M | L | 0 | 1 | 1 | 0 | 0 | 0 | UMUT |
| 16 | Yakushima Island | KGSY-0513 | F | L | 0 | 1 | 1 | 0 | 0 | 0 | UMUT |
| 16 | Yakushima Island | KGSY-0517 | F | L | 0 | 1 | 1 | 0 | 0 | 0 | UMUT |
| 16 | Yakushima Island | M301 | M | L | 0 | 1 | 0 | 1 | 0 | 1 | TPM |
| 16 | Yakushima Island | M302 | M | L | 0 | 1 | 1 | 0 | 0 | 0 | TPM |
| 16 | Yakushima Island | M303 | M | L | 0 | 1 | 0 | 1 | 0 | 1 | TPM |
| 16 | Yakushima Island | M304 | F | L | 0 | 1 | 1 | 0 | 0 | 0 | TPM |
| 16 | Yakushima Island | M305 | M | L | 0 | 1 | 1 | 0 | 0 | 0 | TPM |
| 16 | Yakushima Island | M308 | F | L | 0 | 1 | 0 | 1 | 0 | 1 | TPM |
| 16 | Yakushima Island | M309 | F | L | 0 | 1 | 1 | 0 | 0 | 0 | TPM |
| 16 | Yakushima Island | M311 | F | L | 0 | 1 | 0 | 1 | 0 | 1 | TPM |
| 16 | Yakushima Island | M313 | M | L | 0 | 1 | 1 | 0 | 0 | 0 | TPM |
| 16 | Yakushima Island | M315 | M | L | 0 | 1 | 1 | 0 | 0 | 0 | TPM |
| 16 | Yakushima Island | M316 | M | L | 0 | 1 | 1 | 0 | 0 | 0 | TPM |
| 16 | Yakushima Island | M318 | M | L | 0 | 1 | 0 | 1 | 0 | 1 | TPM |
| 16 | Yakushima Island | M319 | M | L | 0 | 1 | 1 | 0 | 0 | 0 | TPM |

^*^The scoring scheme of mesowear score was based on Croft and Weinstein [27]. ^**^Abbreviations of the institutes are: UMUT, The University Museum, The University of Tokyo; TPM, Tochigi Prefectural Museum; NHMIC, Natural History Museum and Institute, Chiba; HUM, The Hokkaido University Museum.
