# Supplementary material for: The Inter-Relationship between Dietary and Environmental Properties and Tooth Wear: Comparisons of Mesowear, Molar Wear Rate, and Hypsodonty Index of Extant Sika Deer Populations
Source: PLoS One. 2014 Mar 6;9(3):e90745. doi: 10.1371/journal.pone.0090745 (PMC3946258; doi:10.1371/journal.pone.0090745)
Supplement: Table S2 — Synthesized data on mesowear score (MS) and dietary information of 75 ungulate species. (DOC) [file pone.0090745.s004.doc]

Supplementary Table S2. Synthesized data on mesowear score (MS) and dietary information of 75 ungulate species.

| Species | Common name | N | MS | MS (SD) | Mesowear data source^*^ | Graminoid (%) | Non-graminoid (%) | Fruit (%) | Variable | Nature of data | Reliable | References for diet |
| --- | --- | --- | --- | --- | --- | --- | --- | --- | --- | --- | --- | --- |
| *Aepyceros melampus* | Impala | 17 | 0.65 | 0.49 | 1 | 45 | 45 | 10 | Yes | Average | Yes | [1] |
| *Alcelaphus buselaphus* | Hartebeest | 76 | 1.62 | 1.01 | 1 | 75 | 20 | 5 | Yes | Average | Yes | [1] |
| *Alces alces* | Moose | 30 | 0.00 | 0.00 | 1 | 1.3 | 97.2 | 0.0 | No | Average | Yes | [2-12] |
| *Ammodorcas clarkei* | Dibatag | 7 | 0.71 | 0.49 | 1 | 10 | 80 | 10 | No | Estimate | No | [1] |
| *Antidorcas marsupialis* | Springbok | 26 | 0.37 | 0.63 | 1 | 32.5 | 66 | 1.5 | Yes | Average | Yes | [1] |
| *Antilocapra americana* | Pronghorn | 43 | 0.40 | 0.65 | 1 | 8.5 | 91.0 | 0.0 | No | Average | Yes | [13] |
| *Axis axis* | Chital | 43 | 1.47 | 0.96 | 1 | 43.7 | 56.3 | 0.0 | Yes | Average | Yes | [14-16] |
| *Axis porcinus* | Hog deer | 24 | 1.10 | 0.47 | 1 | 66.5 | 8.5 | 6.5 | Unknown | Precise | Yes | [17] |
| *Bison bison* | Bison | 15 | 2.73 | 0.46 | 1 | 93.6 | 3.9 | 0.0 | No | Average | Yes | [18-22] |
| *Boselaphus tragocamelus* | Nilgai | 15 | 1.13 | 0.35 | 1 | 26.2 | 74.0 | 0.0 | No | Average | Yes | [14,15,23] |
| *Budorcas taxicolor* | Takin | 38 | 0.70 | 0.61 | 1 |  |  |  |  |  |  |  |
| *Camelus dromedarius* | Dromedary | 16 | 0.69 | 0.48 | 1 | 7.9 | 92.2 | 0.0 | No | Average | Yes | [24,25] |
| *Capra ibex* | Alpine ibex | 24 | 0.58 | 0.88 | 1 | 60.1 | 39.9 | 0.0 | Unknown | Precise | Yes | [26] |
| *Capreolus capreolus* | Roe deer | 68 | 0.35 | 0.59 | 1 | 5.3 | 84.4 | 1.4 | Yes | Average | Yes | [27] |
| *Capricornis crispus*  (Tochigi population) | Japanese serow | 37 | 0.30 | 0.46 | 3 | 6.5 | 77.7 | 0.0 | Yes | Precise | Yes | [28] |
| *Capricornis crispus*  (Gifu population) | Japanese serow | 30 | 0.53 | 0.51 | 3 | 35.5 | 64.5 | 0.0 | Unknown | Precise | Yes | [29] |
| *Capricornis sumatraensis* | Mainland serow | 22 | 0.77 | 0.69 | 1 |  |  |  |  |  |  |  |
| *Cephalophus dorsalis* | Bay duiker | 28 | 1.00 | 0.67 | 1 | 0 | 27 | 73 | No | Precise | Yes | [1] |
| *Cephalophus natalensis* | Red forest duiker | 6 | 1.00 | 0.00 | 1 | 1 | 20 | 79 | No | Estimate | No | [1] |
| *Cephalophus niger* | Black duiker | 31 | 0.77 | 0.72 | 1 | 1 | 21 | 78 | No | Estimate | No | [1] |
| *Cephalophus nigrifrons* | Black-fronted duiker | 44 | 0.95 | 0.78 | 1 | 0 | 28 | 72 | No | Precise | Yes | [1] |
| *Cephalophus silvicultor* | Yellow-backed duiker | 39 | 1.26 | 0.55 | 1 | 1 | 28 | 71 | No | Precise | Yes | [1] |
| *Ceratotherium simum* | White rhinoceros | 24 | 2.46 | 0.44 | 1 | 99.0 | 1.0 | 0.0 | No | Precise | Yes | [30] |
| *Cervus canadensis* | Wapiti | 19 | 0.53 | 0.51 | 1 | 61.0 | 38.6 | 0.0 | Yes | Average | Yes | [22,31-33] |
| *Cervus duvauceli* | Swamp deer | 50 | 1.49 | 0.98 | 1 | 74.5 | 17.5 | 0.0 | No | Precise | Yes | [17] |
| *Cervus unicolor* | Samber | 21 | 1.07 | 0.64 | 1 | 42.4 | 56.1 | 0.0 | Yes | Average | Yes | [14,15,34] |
| *Connochaetes gnou* | Black wildebeest | 4 | 1.50 | 0.58 | 3 | 81 | 18 | 1 | Yes | Average | Yes | [1] |
| *Connochaetes taurinus* | Blue wildebeest | 52 | 1.64 | 1.09 | 1 | 87.5 | 12 | 0.5 | Yes | Average | Yes | [1] |
| *Damaliscus lunatus* | Tsessebe | 5 | 2.10 | 0.74 | 1 | 95 | 5 | 0 | No | Precise | Yes | [1] |
| *Dendrohyrax arboreus* | Southern tree hyrax | 20 | 0.45 | 0.51 | 1 | 0.0 | 100.0 | 0.0 | No | Average | Yes | [35,36] |
| *Dendrohyrax dorsalis* | Western tree hyrax | 28 | 0.82 | 0.76 | 1 |  |  |  |  |  |  |  |
| *Dicerorhinus sumatrensis* | Sumatran rhinoceros | 5 | 0.20 | 0.45 | 1 | 0.0 | 100.0 | 0.0 | No | Inadequate | No | [30,37] |
| *Diceros bicornis* | Black rinoceros | 34 | 0.06 | 0.24 | 1 | 1.9 | 90.8 | 7.7 | No | Average | Yes | [38-41] |
| *Equus burchelli* | Burchell's zebra | 121 | 2.51 | 0.41 | 1 | 99.4 | 0.6 | 0.0 | No | Average | Yes | [42-44] |
| *Equus grevyi* | Grévy's zebra | 29 | 2.45 | 0.39 | 1 | 100.0 | 0.0 | 0.0 | Unknown | Inadequate | No | [45] |
| *Gazella granti* | Grant's gazelle | 17 | 0.56 | 0.70 | 1 | 65 | 30 | 5 | Yes | Average | Yes | [1] |
| *Gazella thomsonii* | Thomson's gazelle | 146 | 0.63 | 0.79 | 1 | 75 | 20 | 5 | Yes | Average | Yes | [1] |
| *Giraffa camelopardalis* | Giraffe | 61 | 0.36 | 0.65 | 1 | 0.0 | 97.7 | 2.3 | No | Average | Yes | [46-48] |
| *Heterohyrax brucei* | Yellow-spotted hyrax | 11 | 1.64 | 1.14 | 1 | 11.0 | 89.3 | 0.0 | Yes | Precise | Yes | [49] |
| *Hippotragus equinus* | Roan antelope | 26 | 1.12 | 0.43 | 1 | 85 | 10 | 5 | Yes | Average | Yes | [1] |
| *Hippotragus niger* | Sable antelope | 20 | 1.30 | 0.73 | 1 | 85 | 10 | 5 | Yes | Average | Yes | [1] |
| *Hyaemoschus aquaticus* | Water chevrotain | 18 | 0.83 | 0.38 | 1 | 0.0 | 51.2 | 48.8 | Unknown | Presice | Yes | [50] |
| *Kobus ellipsiprymnus* | Waterbuck | 22 | 1.05 | 0.21 | 1 | 84 | 15 | 1 | Yes | Average | Yes | [1] |
| *Kobus kob* | Kob | 8 | 1.00 |  | 2 | 95 | 5 | 0 | No | Inadequate | No | [1] |
| *Kobus leche* | Lechwe | 3 | 1.00 |  | 2 | 95 | 5 | 0 | No | Inadequate | No | [1] |
| *Lama glama* | Llama | 32 | 0.75 | 0.62 | 1 | 88.5 | 11.5 | 0.0 | No | Presice | Yes | [51] |
| *Lama vicugna* | Vicuña | 12 | 0.58 | 0.51 | 1 | 79.6 | 14.3 | 5.4 | No | Average | Yes | [52,53] |
| *Litocranius walleri* | Gerenuk | 69 | 0.71 | 0.55 | 1 | 0 | 95 | 5 | No | Estimate | Yes | [1] |
| *Odocoileus hemionus* | Mule deer | 33 | 0.52 | 0.51 | 1 | 7.3 | 80.8 | 10.8 | No | Average | Yes | [22,33,54-57] |
| *Odocoileus virginianus* | White-tailed deer | 18 | 0.11 | 0.32 | 1 | 5.3 | 85.3 | 7.6 | Yes | Average | Yes | [4,6,54,55,58-63] |
| *Okapia johnstoni* | Okapi | 8 | 0.13 | 0.35 | 1 | 0.0 | 100.0 | 0.0 | No | Inadequate | No | [64] |
| *Oreotragus oreotragus* | Klipspringer | 1 | 1.00 |  | 2 | 20 | 60 | 20 | Yes | Average | Yes | [1] |
| *Oryx gazella* | Gemsbok | 3 | 1.00 |  | 2 | 75 | 20 | 5 | Yes | Average | Yes | [1] |
| *Ourebia ourebi* | Oribi | 128 | 0.85 | 0.51 | 1 | 90 | 10 | 0 | Yes | Estimate | Yes | [1] |
| *Ovibos moschatus* | Muskox | 52 | 0.80 | 0.85 | 1 | 53.6 | 43.9 | 0.0 | Yes | Average | Yes | [65-67] |
| *Ovis canadensis* | Bighorn sheep | 29 | 0.64 | 0.77 | 1 | 44.8 | 55.1 | 0.0 | Yes | Average | Yes | [68-70] |
| *Procavia capensis* | Rock hyrax | 24 | 1.54 | 0.93 | 1 | 61.0 | 40.1 | 0.0 | Yes | Precise | Yes | [49] |
| *Rangifer tarandus* | Reindeer | 27 | 1.96 |  | 2 | 41.4 | 56.8 | 0.2 | Yes | Average | Yes | [71-73] |
| *Raphicerus campestris* | Steenbok | 10 | 1.20 |  | 2 | 34 | 61 | 5 | Yes | Average | Yes | [1] |
| *Raphicerus melanotis* | Cape grysbok | 6 | 1.00 |  | 2 | 30 | 50 | 20 | Yes | Estimate | Yes | [1] |
| *Redunca arundinum* | Southern reedbuck | 1 | 1.00 |  | 2 | 95 | 2.5 | 2.5 | No | Estimate | Yes | [1] |
| *Redunca fulvorufula* | Mountain reedbuck | 7 | 1.14 | 0.38 | 1 | 95 | 2.5 | 2.5 | No | Estimate | Yes | [1] |
| *Redunca redunca* | Bohor reedbuck | 77 | 1.07 | 0.48 | 1 | 95 | 5 | 0 | No | Estimate | Yes | [1] |
| *Rinoceros sondaicus* | Javan rhinoceros | 5 | 0.00 | 0.00 | 1 | 0.0 | 98.9 | 1.1 | No | Inadequate | No | [74] |
| *Rinoceros unicornis* | Indian rhinoceros | 5 | 0.20 | 0.45 | 1 | 63.4 | 30.0 | 0.3 | Yes | Average | Yes | [17,75,76] |
| *Saiga tatarica* | Saiga | 5 | 1.60 | 1.08 | 1 | 28.1 | 42.1 | 0.0 | Yes | Average | Yes | [77,78] |
| *Sigmoceros lichtensteinii* | Lichtenstein's hartebeest | 17 | 1.32 | 0.73 | 1 | 91.9 | 8.1 | 0.0 | No | Average | Yes | [42,79] |
| *Syncerus caffer* | African buffalo | 31 | 1.13 | 0.50 | 1 | 77.5 | 22.5 | 0 | Yes | Average | Yes | [1] |
| *Taurotragus oryx* | Eland | 14 | 0.50 | 0.52 | 1 | 50 | 45 | 5 | Yes | Average | Yes | [1] |
| *Tetracerus quadricornis* | Four-horned antelope | 21 | 0.88 | 0.63 | 1 | 9.4 | 90.5 | 0.0 | Unknown | Precise | Yes | [23] |
| *Tragelaphus angasii* | Nyala | 20 | 0.65 | 0.49 | 1 | 20 | 50 | 30 | Yes | Average | Yes | [1] |
| *Tragelaphus eurycerus* | Bongo | 27 | 0.59 | 0.50 | 1 | 8.8 | 90.0 | 1.3 | No | Average | Yes | [80,81] |
| *Tragelaphus imberbis* | Lesser kudu | 31 | 0.42 | 0.50 | 1 | 33.5 | 64 | 2.5 | Yes | Average | Yes | [1] |
| *Tragelaphus scriptus* | Bushbuck | 47 | 0.51 | 0.51 | 1 | 10 | 80 | 10 | Yes | Average | Yes | [1] |
| *Tragelaphus spekii* | Sitatunga | 8 | 0.75 | 0.46 | 3 | 67.5 | 30 | 2.5 | Yes | Estimate | Yes | [1] |
| *Tragelaphus strepsiceros* | Grearter kudu | 7 | 1.00 | 0.00 | 1 | 15 | 55 | 30 | Yes | Estimate | Yes | [1] |

^*^The sources of the mean and standard deviation (SD) of the mesowear score were (1) calculation from the original data set from the study by Fortelius and Solounias [82]; (2) data from the study by Kaiser et al. [83] (SD not presented); and (3) data from the present study.
