# Supplementary material for: The Inter-Relationship between Dietary and Environmental Properties and Tooth Wear: Comparisons of Mesowear, Molar Wear Rate, and Hypsodonty Index of Extant Sika Deer Populations
Source: PLoS One. 2014 Mar 6;9(3):e90745. doi: 10.1371/journal.pone.0090745 (PMC3946258; doi:10.1371/journal.pone.0090745)
Supplement: Table S3 — Selection of the best model predicting MS, the molar wear rate or the M3 hypsodonty index of the Japanese sika deer. The best models with the lowest AICc value are in bold font. In the case where the M3 hypsodonty index was the response variable, the model with the lowest AICc value included both graminoid proportion and annual precipitation. However, annual precipitation was not statistically significant (P = 0.06). Therefore, we selected the model included only graminoid proportion as the best model for the M3 hypsodonty index. The predictor variables are: G = graminoid proportion; F = fruit proportion; AP = annual precipitation; G×AP = interaction between graminoid proportion and annual precipitation; F×AP = interaction between fruit proportion and annual precipitation. K = number of parameters in the model; AICc = Akaike information criterion corrected for sample size; + = factor included in the model. (DOC) [file pone.0090745.s005.doc]

Supplementary Table S3. Selection of the best model predicting MS, the molar wear rate or the M_3_ hypsodonty index of the Japanese sika deer. The best models with the lowest AICc value are in bold font. In the case where the M_3_ hypsodonty index was the response variable, the model with the lowest AICc value included both graminoid proportion and annual precipitation. However, annual precipitation was not statistically significant (*P* = 0.06). Therefore, we selected the model included only graminoid proportion as the best model for the M_3_ hypsodonty index. The predictor variables are: G = graminoid proportion; F = fruit proportion; AP = annual precipitation; G × AP = interaction between graminoid proportion and annual precipitation; F × AP = interaction between fruit proportion and annual precipitation. K = number of parameters in the model; AICc = Akaike information criterion corrected for sample size; + = factor included in the model.

|  | Predictor variable in the model | | | | |  |  |
| --- | --- | --- | --- | --- | --- | --- | --- |
| Response variable | G | F | AP | G × AP | F × AP | K | AICc |
| **MS** | **+** |  |  |  |  | **1** | **-5.27** |
| MS | + |  | + |  |  | 2 | -1.69 |
| MS | + |  | + | + |  | 3 | 2.96 |
| MS | + | + | + | + |  | 4 | 8.79 |
| MS | + | + | + | + | + | 5 | 16.08 |
| MS |  | + |  |  |  | 1 | 2.28 |
| MS |  | + | + |  |  | 2 | 5.14 |
| MS |  | + | + |  | + | 3 | 9.81 |
| MS | + | + | + |  | + | 4 | 8.66 |
| MS | + | + | + |  |  | 3 | 2.97 |
| MS | + | + |  |  |  | 2 | -1.45 |
|  |  |  |  |  |  |  |  |
| **Molar wear rate** | **+** |  |  |  |  | **1** | **-155.98** |
| Molar wear rate | + |  | + |  |  | 2 | -152.35 |
| Molar wear rate | + |  | + | + |  | 3 | -148.94 |
| Molar wear rate | + | + | + | + |  | 4 | -145.53 |
| Molar wear rate | + | + | + | + | + | 5 | -138.72 |
| Molar wear rate |  | + |  |  |  | 1 | -131.34 |
| Molar wear rate |  | + | + |  |  | 2 | -129.01 |
| Molar wear rate |  | + | + |  | + | 3 | -124.35 |
| Molar wear rate | + | + | + |  | + | 4 | -145.80 |
| Molar wear rate | + | + | + |  |  | 3 | -150.17 |
| Molar wear rate | + | + |  |  |  | 2 | -154.76 |
|  |  |  |  |  |  |  |  |
| **M_3_ hypsodonty index** | **+** |  |  |  |  | **1** | **-37.76** |
| M_3_ hypsodonty index | + |  | + |  |  | 2 | -38.68 |
| M_3_ hypsodonty index | + |  | + | + |  | 3 | -34.14 |
| M_3_ hypsodonty index | + | + | + | + |  | 4 | -28.66 |
| M_3_ hypsodonty index | + | + | + | + | + | 5 | -24.23 |
| M_3_ hypsodonty index |  | + |  |  |  | 1 | -32.83 |
| M_3_ hypsodonty index |  | + | + |  |  | 2 | -29.60 |
| M_3_ hypsodonty index |  | + | + |  | + | 3 | -25.09 |
| M_3_ hypsodonty index | + | + | + |  | + | 4 | -29.88 |
| M_3_ hypsodonty index | + | + | + |  |  | 3 | -34.49 |
| M_3_ hypsodonty index | + | + |  |  |  | 2 | -34.61 |
