# Supplementary material for: The Inter-Relationship between Dietary and Environmental Properties and Tooth Wear: Comparisons of Mesowear, Molar Wear Rate, and Hypsodonty Index of Extant Sika Deer Populations
Source: PLoS One. 2014 Mar 6;9(3):e90745. doi: 10.1371/journal.pone.0090745 (PMC3946258; doi:10.1371/journal.pone.0090745)
Supplement: Table S4 — Comparative data on the molar wear rate, the M3 hypsodonty index, and ecological variables for nine living ruminants. (DOC) [file pone.0090745.s006.doc]

Supplementary Table S4. Comparative data on the molar wear rate, the M_3_ hypsodonty index, and ecological variables for nine living ruminants.

| Species | Proportion of graminoids in diet (%)^*^ | Annual precipitation (mm)^**^ | Molar wear rate^***^ | M_3_ hypsodonty index^***^ |
| --- | --- | --- | --- | --- |
| 1. *Antidorcas marsupialis* | 33 | 24.00 | 1.41 | 4.89 |
| 2. *Bison bison bison* | 94 | 33.59 | 3.65 | 4.87 |
| 3. *Capreolus capreolus* | 5 | 55.04 | 0.33 | 1.49 |
| 4. *Cervus elaphus* | 61 | 44.42 | 0.93 | 2.11 |
| 5. *Connochaetes taurinus* | 88 | 61.61 | 2.03 | 4.94 |
| 6. *Gazella granti* | 65 | 58.07 | 1.63 | 3.45 |
| 7. *Ovibos moschatus* | 54 | 15.78 | 3.51 | 3.69 |
| 8. *Rangifer tarandus* | 36 | 29.21 | 0.92 | 1.52 |
| 9. *Syncerus caffer caffer* | 78 | 100.45 | 2.77 | 3.00 |

^*^From this study (data presented in Supplementary Table S1). ^**^From the study by Kaiser et al. (2013). ^***^From the study by Solounias et al. (1994).
