# Supplementary material for: The Inter-Relationship between Dietary and Environmental Properties and Tooth Wear: Comparisons of Mesowear, Molar Wear Rate, and Hypsodonty Index of Extant Sika Deer Populations
Source: PLoS One. 2014 Mar 6;9(3):e90745. doi: 10.1371/journal.pone.0090745 (PMC3946258; doi:10.1371/journal.pone.0090745)
Supplement: Table S5 — Estimation of silica content in deer diet. Estimated silica content (%) is further used for regression analyses testing the relationship between silica content and dental variables (See Fig. S2). (DOC) [file pone.0090745.s007.doc]

Supplementary Table S5. Estimation of silica content in deer diet. Estimated silica content (%) is further used for regression analyses testing the relationship between silica content and dental variables (See Fig. S2).

| Population |  | Mean annual  consumption (%) | Si (%)^a^ | Estimated silica in diet (%)^b^ |
| --- | --- | --- | --- | --- |
| 1. Hokkaido | Graminoids | 18.8 |  | **0.79** |
|  | *Sasa nipponica* | 13.7 | 4.32 | 0.59 |
|  | *Carex* spp. | 3.4 | 1.31^c^ | 0.04 |
|  | Pasture grass (*Dactylis glomerata*) | 27.6 | 0.56 | 0.15 |
|  | Others | 2.0 |  |  |
| 2. Mt. Goyo | Graminoids  (*Sasa nipponica*) | 52.5 | 4.32 | **2.27** |
| 3. Kinkazan | Graminoids | 65.8 |  | **0.71**^d^ |
|  | *Zoysia japonica* | 22.3 | 1.33 | 0.30 |
|  | *Miscanthus sinensis* | 5.0 | 2.96 | 0.15 |
|  | *Brachypodium sylvaticum* | 3.0 | 3.06 | 0.09 |
|  | *Poa annua* | 3.8 | 1.36^e^ | 0.05 |
|  | *Agrostis clavata* | 3.1 | 2.26^f^ | 0.07 |
|  | *Festuca rubra* | 3.3 | 1.52 | 0.05 |
|  | Other graminoids | 10.0 |  | NA |
|  | Culms and sheaths | 15.3 |  | NA |
| 4. Nikko | Graminoids | 73.9 |  | **2.83** |
|  | *Sasa nipponica* | 59.3 | 4.32 | 2.56 |
|  | Other grasses  (*Calamagrostis sachalinensis*,  *Festuca rubra*) | 12.0 | 1.89^g^ | 0.23 |
|  | Sedges | 2.9 | 1.31^c^ | 0.04 |
| 5. Ashio | Graminoids  (*Miscanthus sinensis*) | 90.0 | 2.96 | **2.66** |
| 6. Okutama | Graminoids  (*Sasa nipponica*) | 32.1 | 4.32 | **1.39** |
| 7. Boso | Graminoids  (*Carex* spp.) | 45.2 | 1.31^c^ | **0.59** |
| 8. Izu | Graminoids  (*Sasa nipponica*) | 30.5 | 4.32 | **1.32** |
| 9. Yamanashi | Graminoids  (*Sasa nipponica*) | 42.2 | 4.32 | **1.82** |
| 10. Nara | Graminoids  (*Zoysia japonica*) | 81.4 | 1.33 | **1.08** |
| 11. Shimane | Graminoids | 38.1 |  | **0.74** |
|  | *Carex* spp. | 31.9 | 1.31^c^ | 0.42 |
|  | *Pleioblastus chino* | 6.2 | 5.17 | 0.32 |
| 12. Yamaguchi | Graminoids  (*Pleioblastus chino*) | 6.5 | 5.17 | **0.34** |
| 13. Fukuoka | Graminoids (undescribed) | 7.2 |  | **NA** |
| 14. Mt. Shiraga | Graminoids  (*Carex* spp.) | 25.6 | 1.31^c^ | **0.34** |
| 15. Tsushima | Graminoids  (*Carex* spp., *Panicum* spp.) | 3.4 | 1.81^h^ | **0.06** |
| 16. Yakushima | Graminoids (undiscribed) | 4.4 |  | **NA** |

^a^Silicon (Si) concentration in plant shoot was obtained from Hodson et al. [5] (reference number is the same in the reference list of the main text). ^b^Estimated silica content in sika deer diet was calculated by the following fomula:

$$\text{Estimated silica in deer diet }=\text{ }\sum\text{(mean annual consumption of each graminoid species)}\times(\text{Si concentration of each graminoid species)}.$$

^c^Average value of 13 species of genus *Carex*. ^d^Since contribution of “Other graminoids” and “Culms and sheaths” is large in deer diet (10.1 and 15.3%, respectively), this value underestimates the actual value of silica content in diet of Kinkazan deer. ^e^Average value of 4 species of genus *Poa*. ^f^Average value of 6 species of genus *Agrostis*. ^g^Mean value of *Calamagrostis sachalinensis* (2.25%) and *Festuca rubra* (1.52%). ^h^Mean value of the genus *Carex* (1.31%) and the genus *Panicum* (2.30%).
